# miR-147b mediated suppression of DUSP8 promotes lung cancer progression

**DOI:** 10.1038/s41388-024-02969-7

**Published:** 2024-02-23

**Authors:** Kati Turkowski, Frederik Herzberg, Stefan Günther, Andreas Weigert, Tamara Haselbauer, Ludger Fink, David Brunn, Friedrich Grimminger, Werner Seeger, Holger Sültmann, Thorsten Stiewe, Soni S. Pullamsetti, Rajkumar Savai

**Affiliations:** 1 Max Planck Institute for Heart and Lung Research, Member of the German Center for Lung Research (DZL), Member of the Cardio-Pulmonary Institute (CPI), Bad Nauheim, 61231 Germany; 2 Institute for Lung Health (ILH), Justus Liebig University, 35392 Giessen, Germany; 3 Goethe-University Frankfurt, Faculty of Medicine, Institute of Biochemistry I, Frankfurt, Germany; 4 Frankfurt Cancer Institute (FCI), Goethe University, and German Cancer Consortium (DKTK), Hesse, Germany; 5 Institute of Pathology and Cytology, UEGP, Wetzlar, Germany; 6 Department of Internal Medicine, Member of the DZL, Member of CPI, Justus Liebig University, 35392 Giessen, Germany; 7 Cancer Genome Research Group, German Cancer Research Center (DKFZ), Germany Center for Lung Research (DZL), and German Cancer Consortium (DKTK), Heidelberg, Germany; 8 Institute of Molecular Oncology, Philipps-University, 35043 Marburg, Germany

**Keywords:** Non-small-cell lung cancer, Non-coding RNAs, Phosphorylation

## Abstract

Dual-specificity phosphatase 8 (DUSP8) plays an important role as a selective c-Jun N-terminal kinase (JNK) phosphatase in mitogen-activated protein kinase (MAPK) signaling. In this study, we found that DUSP8 is silenced by miR-147b in patients with lung adenocarcinoma (LUAD), which correlates with poor overall survival. Overexpression of DUSP8 resulted in a tumor-suppressive phenotype in vitro and in vivo experimental models, whereas silencing DUSP8 with a siRNA approach abrogated the tumor-suppressive properties. We found that miR-147b is a posttranscriptional regulator of DUSP8 that is highly expressed in patients with LUAD and is associated with lower survival. NanoString analysis revealed that the MAPK signaling pathway is mainly affected by overexpression of miR-147b, leading to increased proliferation and migration and decreased apoptosis in vitro. Moreover, induction of miR-147b promotes tumor progression in vitro and in vivo experimental models. Knockdown of miR-147b restored DUSP8, decreased tumor progression in vitro, and increased apoptosis via JNK phosphorylation. These results suggest that miR-147b plays a key role in regulating MAPK signaling in LUAD. The link between DUSP8 and miR-147b may provide novel approaches for the treatment of lung cancer.

## Introduction

Dysregulation of mitogen-activated protein kinase (MAPK) signaling plays a crucial role in the progression of lung cancer and several other cancer types [1, 2]. The c-Jun N-terminal kinase (JNK) pathway is one of three well-characterized MAPK pathways involved in numerous cellular functions in tumor development including proliferation, differentiation, survival, and apoptosis. Protein kinases transfer the terminal phosphate group of adenosine triphosphate (ATP) to catalyze specific amino acid residues in substrates, thereby altering the functions of target proteins, either by affecting their activities or controlling their subcellular localization [3]. In addition, protein kinases can be self-activated by auto-phosphorylation or phosphorylation of other kinases through the addition of phosphate groups to their specific amino acid residues [3]. Like other MAPK proteins, JNK proteins are activated by a series of phosphorylation events. The MAP2K protein kinases (e.g., MKK4 and MKK7) phosphorylate JNKs directly at threonine-183 and tyrosine-185 and are in turn activated by dual phosphorylation of MAP3Ks. In addition, JNKs regulate kinase activity through interaction with scaffold proteins as well as dual-specific phosphatases (DUSPs) and nuclear factor kappa B (NF-κB) [4].

Increasing evidence shows that DUSPs are deregulated in cancer, highlighting the importance of these kinases in cancer progression [5]. Unlike other DUSPs, DUSPs such as DUSP8, DUSP10, and DUSP16, which are a subfamily of DUSPs, are located in cytoplasmic and nuclear compartments and contain a kinase-interacting motif (KIM) that interacts with the common docking domain of MAPKs to mediate the enzyme-substrate interaction. This subfamily of DUSPs is relatively selective in terms of dephosphorylation of the phosphorylated serine, threonine, and tyrosine residues of the p38, ERK, and JNK substrates [6]. In particular, DUSP8 preferentially dephosphorylates relevant residues of JNK [7, 8]. There are several ways to regulate the activation of DUSP8, including protein stability, gene transcription, and phosphatase activity. DUSP8 expression can be rapidly induced via oxidative stress, heat shock, growth factors, or small-molecule activators [9, 10]. Besides transcriptional regulation, another important part of DUSP8 expression is post-transcriptional regulation. So far, however, the role of DUSP8 in cancer and the molecules involved in the post-transcriptional regulation of DUSP8 remain barely investigated. Previous studies reported that miRNAs, which are a class of important gene post-transcriptional factors, may be involved in post-transcriptional regulation by binding to the 3′-untranslated region (3′-UTR) of DUSP8 and thereby inhibiting the suppressive effect of DUSP8 on colorectal cancer cell proliferation and migration [11] and MAPK inhibitor resistance [12, 13].

In this study, we examined the functional impact of DUSP8 in lung cancer progression using in vitro and in vivo models. We also analyzed the post-transcriptional regulation of DUSP8 via miR-147b and the oncogenic potential of this miRNA using in vitro and in vivo models to determine its biological function in lung cancer progression.

## Results

### DUSP8 downregulation is associated with poor overall survival in patients with LUAD

**Fig. 1:**
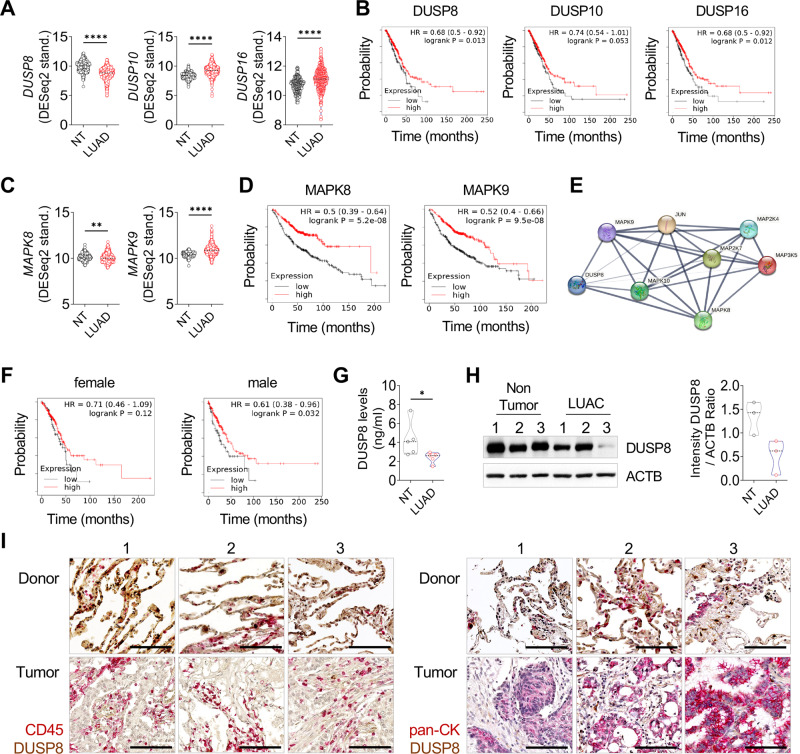
Downregulation of *DUSP8* in LUAD correlated with poor OS. **A** mRNA expression analysis of *DUSP8, DUSP10*, and *DUSP16* in samples obtained from patients with LUAD (*n* = 513) compared to non-tumor tissues (NTs) (*n* = 287) from the GTEx–TCGA patient cohort. **B** The clinical outcome associated with DUSP8, DUSP10, and DUSP16 expression in LUAD using KM Plotter [36]. **C** Expression of *MAPK8* and *MAPK9* in LUAD (*n* = 513) vs. NT (*n* = 287) respectively. **D** Correlation between OS of patients with LUAD and expressions of *MAPK8* and *MAPK9*. **E** Protein interaction network using STRING. The edges indicate both functional and physical protein associations, line thickness indicates the strength of data support. **F** Correlation between OS of female and male patients in all stages of LUAD and DUSP8 expression. **G** DUSP8 protein level measured using ELISA (*n* = 5). **H** Western blot analysis of DUSP8 expression in NTs and tumor samples from the same patient (*n* = 3). **I** Representative images of DUSP8 (brown) co-staining with CD45 or pan-CK (red), in LUAD and healthy donor tissue samples, (*n* = 3), scale bar 50 µm. Data are presented as mean ± standard error of the mean using a two-tailed unpaired t-test with Welch’s correction. For all analyses, *P*-values ≤ 0.05 were considered statistically significant. *p ≤ 0.05, **p ≤ 0.01, and ****p ≤ 0.0001.

To dissect the role of MAPs in LUAD, we first performed in silico analysis of the most common group of DUSPs (e.g., DUSP8, DUSP10, DUSP16). Interestingly, we found a significant, exclusive downregulation of the mRNA expression of *DUSP8* in the LUAD GTEx-TCGA patient cohort compared to the normal tissue (Fig. 1A). A positive correlation was observed between *DUSP8* expression and overall survival (OS) in patients with LUAD (Fig. 1B). Further screening for the mRNA expression of the MAPK pathway genes *MAPK8 and MAPK9* in the patient cohort revealed significantly downregulated expressions of MAPK8 while MAPK9 is upregulated in LUAD patients (Fig. 1C), depicting an association of MAPK8 expression with decreased OS (Fig. 1D). In addition, *DUSP8* expression correlated negatively with *MAPK8* expression but correlated positively with *MAPK9* expression (Supplementary Fig. S1A), while proteomic data [14] showed decreased protein expression (Supplementary Fig. S1B) and less expression of phosphorylation sites for both JNK1 and JNK2 in LUAD patients (Supplementary Fig. S1C). A protein interaction network created with STRING showed strong relationships between DUSP8 and the MAP kinases associated with JNK signaling, whose cascade is known to play a role in apoptosis [15, 16] (Fig. 1E). An additional in silico analysis revealed differences in OS upon DUSP8 expression in all LUAD stages between female and male especially in regard to stage and mutation burden (Fig. 1F and Supplementary Fig. S1D–F). In addition, reduced levels of DUSP8 were detected in protein samples from patients with LUAD (Fig. 1G, H) and IHC staining, where DUSP8 is mainly expressed by alveolar cells, not in leukocytes (Fig. 1I). We also found that the JNK upstream molecules MAP2K7 and MAP3K5 were downregulated in patients with LUAD and correlated negatively with clinical outcome (Supplementary Fig. S1G, H). Moreover, *DUSP8* is not exclusively downregulated in LUAD, but it is also downregulated in pancreatic ductal adenocarcinoma (PDAC), kidney renal papillary cell carcinoma (RPCC), and cervical squamous cell carcinomas (CSCC) (Supplementary Fig. S1I), again being associated with poor OS (Supplementary Fig. S1J).

### Modulation of DUSP8 expression suppresses or supports cancer progression in vitro

**Fig. 2:**
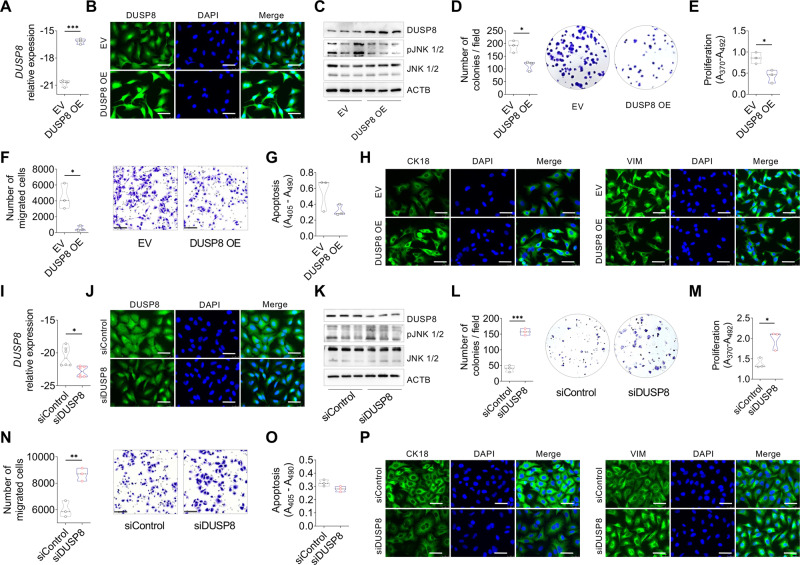
Upregulation of DUSP8 leads to a suppressive phenotype, whereas down-regulation of DUSP8 is tumor-promoting in vitro. Validation of DUSP8 overexpression after transfection of A549 cells with empty vector (EV) and DUSP8 expression vector (OE) was quantified using (**A**) qRT-PCR (*n* = 3), (**B**) immunofluoresence staining, and (**C**) western blot (*n* = 3). Phosphorylation of JNK was performed using a western blot of A549-EV and A549-DUSP8 OE cells (*n* = 3). **D** Comparison of colony formation between DUSP8 OE cells and EV control cells (*n* = 3). **E** Comparison of cellular proliferation between DUSP8 OE cells and EV control cells using BrdU assay (*n* = 3). **F** Migratory ability of DUSP8 OE cells assessed using Boyden chamber assay (*n* = 3). **G** Apoptosis of DUSP8 OE cells compared to EV control cells (*n* = 3). **H** Representative immunofluorescence images of EMT markers expression using antibodies against CK18 and VIM (green) counterstained with DAPI (blue), scale bars, 50 µm. **I** mRNA expression of *DUSP8* after siRNA transfection with DUSP8 siRNA and non-targeting siRNA control (siNT) (*n* = 6). **J** Representative immunocytochemistry images of DUSP8 after treatment with DUSP8 siRNA compared to a non-targeting control. Cells in panels **B** and **J** were labeled using DUSP8 antibody and revealed by Alexa Fluor 488 secondary antibody (green). DNA was stained with DAPI (blue) (scale bars: 50 µm). **K** Western blotting of A549 cells after treatment with DUSP8 siRNA (*n* = 3). Functional assessment via **L** colony formation, **M** cell proliferation, **N** migration and **O** apoptosis of A549 cells after treatment with DUSP8 siRNA. **P** Representative immunofluorescence images of EMT marker expression using antibodies against CK18 and VIM (green) counterstained with DAPI (blue), scale bars, 50 µm. Data are shown as mean ± standard error of the mean using a two-tailed unpaired t-test with Welch’s correction. *P*-values ≤ 0.05 were considered statistically significant for all analyses, *p ≤ 0.05, **p ≤ 0.01, ***p ≤ 0.001 and ***p ≤ 0.0001.

Based on the above findings, we overexpressed or silenced DUSP8 in human lung cancer cell lines to investigate its role in lung cancer progression. A549 and H1299 cells were transfected with a vector overexpressing DUSP8 (OE) or an empty vector (EV) used as control, and the cells were assessed at the mRNA (Fig. 2A and Supplementary Fig. S2A) and protein levels (Fig. 2B, C, Supplementary Figs. S3A and S2B, C). In addition, we performed nuclear and cytoplasmic fractionation of proteins isolated from NSCLC cell lines (A549 and H1299) and DUSP8 overexpressing cell lines to evaluate the distribution of the protein (Supplementary Fig. S3B, C). To determine the impact of DUSP8 overexpression on the phosphorylation of MAP kinase-specific substrates, we further analyzed the protein expression of a known DUSP8-specific substrate (i.e., JNK). The reduction of phospho-JNK in overexpressing cells was compared to that in EV cells (Fig. 2C, Supplementary Figs. S2C and S3D). Further, detection of upstream JNK signaling MAP kinases (Supplementary Fig. S3E) revealed no significant changes in phosphorylation upon DUSP8 overexpression, except phosphorylated JNK (Supplementary Fig. S3D–G). In addition, a phosphokinase array was performed to determine other possible substrates of DUSP8, indicating reduced phosphorylation of kinase phosphorylation sites of e.g. GSK-3α/β, GSK-3β, Src, STAT5a/b, WNK1, PRAS40, RSK1/2 and decreased phosphorylation of β-catenin, c-Jun and HSP60 in protein lysates of A549-DUSP8-overexpressing cells (Supplementary Fig. S3H). Functional assays revealed overexpressing cell lines had lower colony-forming ability than EV control cells (Fig. 2D and Supplementary Fig. S2D). The assays also showed that DUSP8 OE cells had significantly less proliferation and migration than EV control cells (Fig. 2E, F, Supplementary Fig. S2E, F). Further analysis revealed no significant effect on apoptosis by DUSP8 overexpression (Fig. 2G and Supplementary Fig. S2G). We also observed changes in epithelial to mesenchymal transition (EMT) marker expression of DUSP8 overexpressing cells via upregulation of cytokeratin (CK18) and downregulation of vimentin (VIM) (Fig. 2H, Supplementary Figs. S3I and S2H, I). The same experiments were performed vice versa using siRNA against DUSP8. Therefore, we analyzed the expression of DUSP8 in different NSCLC cell lines (A549, H838, H1299 and H1650) and selected the cell lines for silencing based on the basal expression level of DUSP8, which was significantly higher in H838 cells than in A549, H1299 and H1650 cells (Supplementary Fig. S3J). The silencing of DUSP8 showed the opposite effect in A549 and H838 cells with decreased expression of DUSP8 (Fig. 2I–K and Supplementary Fig. S4A–C), with increased phosphorylated JNK (Fig. 2K, Supplementary Figs. S3K and S4C) and significantly increased proliferation, colony formation and migration (Fig. 2L–N, and Supplementary Fig. S4D–F). In addition, apoptosis was not affected by DUSP8 silencing (Fig. 2O and Supplementary Fig. S4G), however the expression of the EMT markers CK18 and VIM changed (Fig. 2P, Supplementary Figs. S3L and S4H, I).

### Increased DUSP8 expression suppresses cancer growth in mice in vivo

**Fig. 3:**
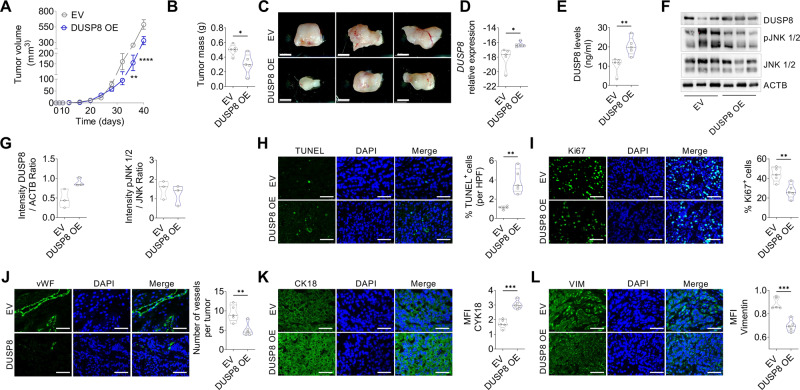
Impact of DUSP8 overexpression on cancer progression in vivo. A549-EV and A549-DUSP8 OE cells were injected into the right flank of immunodeficient mice. Tumors were harvested after 40 days. **A** Measurement of tumor size during tumor progression (*n* = 5). **B** Measurement of tumor mass after 40 days (*n* = 5). **C** Representative photographs of dissected DUSP8 overexpressing tumors, scale bar 5 mm. Validation of DUSP8 expression in mice tumor tissue samples via **D** mRNA (*n* = 5), **E** protein using ELISA (*n* = 5), and **F** western blot and **G** quantification of band intensity (*n* = 3). **H** Representative photomicrographs of TUNEL staining for apoptotic cells within the tumor counted per high power field (HPF) using Fiji Software (*n* = 5, 4 images per animal). **I** Representative photomicrographs of Ki67 staining of proliferating cells within the tumor counted per HPF using Fiji Software (*n* = 5, 5 images per animal). **J** Representative photomicrographs of vascular marker von Willebrand factor (vWF) quantified per HPF using Fiji (*n* = 5, 5 images per animal). Representative photomicrographs of EMT marker (**K**) CK18 and (**L**) VIM quantified via calculation of the mean fluorescent intensity (MFI) using Fiji (*n* = 5, 3 images per animal). Ki67, vWF, CK18, and VIM staining were visualized using Alexa Flour 488 coupled secondary antibody (green). Nuclear DNA was counterstained with DAPI (blue), scale bar 50 µm. Data are shown as mea*n* ± standard error of the mean using two-way ANOVA with Bonferroni’s multiple comparisons (**A**) and two-tailed unpaired t-test with Welch’s correction (**B**–**L**). *P*-values ≤ 0.05 were considered statistically significant for all analyses, *p ≤ 0.05, **p ≤ 0.01 and **p ≤ 0.001.

To determine the tumor-suppressive properties of DUSP8 overexpressing cells in vivo, we transplanted A549-DUSP8 OE and A549-EV cells subcutaneously into the right flank of NOD.Cg-Prkdcscid Il2rgtm1Wjl/SzJ (NSG) immunodeficient mice. Tumor progression was measured using calipers every fourth day until 40 days (313.24 ± 103.41 mm^3^ vs. 181.56 ± 58.22 mm^3^). The increase in tumor volume and mass was significantly decreased upon use of A549-DUSP8 OE as compared to A549-EV cells (Fig. 3A, B), and the decreased tumor progression was observable macroscopically after tumor harvest (Fig. 3C). Notably, we could detect increased DUSP8 expression at the mRNA (Fig. 3D) and protein levels (Fig. 3E–G) in the tumor tissue after 40 days of tumor growth. In addition, JNK showed a tendency towards reduced phosphorylation in the tissue samples (Fig. 3F, G). Furthermore, DUSP8 overexpressing tumors revealed a significantly increased number of apoptotic cells (Fig. 3H) in the tumor area and decreased numbers of proliferating cells (Fig. 3I) and blood vessels (Fig. 3J) in the tissue samples compared to the EV-inoculated control group. Moreover, immunohistochemistry stainings of the DUSP8 tumor-bearing tissues showed increased expression of cytokeratin (Fig. 3K) while vimentin (Fig. 3L) was downregulated. These findings confirm the tumor-suppressive properties of DUSP8.

### DUSP8 silencing is mediated by upregulation of miR-147b

**Fig. 4:**
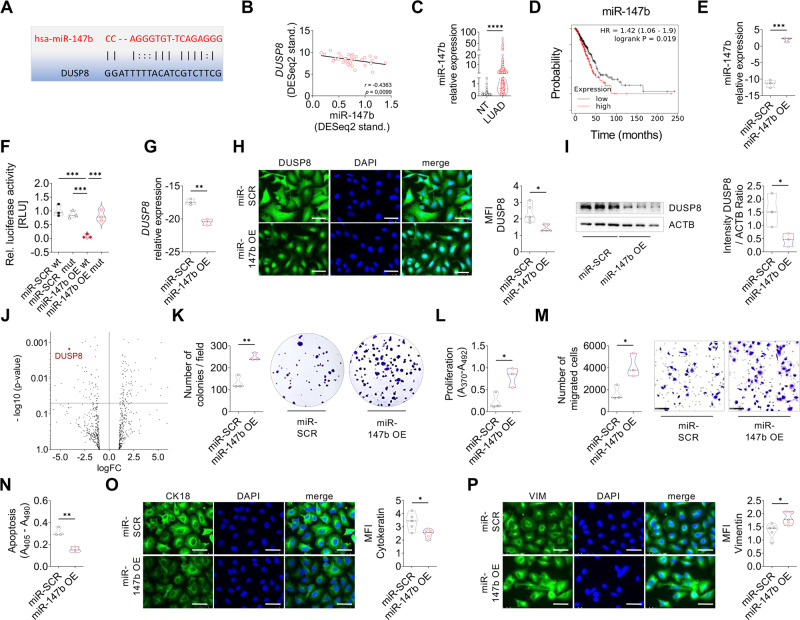
High expression of miR-147b promotes tumor progression in vitro and is correlated with low OS in lung cancer. **A** miR-147b - DUSP8 binding site predicted via RNA22. **B** Scatter plots of *DUSP8* expression correlated with miR-147b expression in LUAD samples from the TCGA dataset (*n* = 34). The *r*-value and two-tailed, *p*-value were calculated using Pearson’s rank correlation coefficients. **C** In silico analysis of miR-147b expression in LUAD (*n* = 458) vs. non-tumor tissue (*n* = 46) from the same TCGA cohort. **D** Correlation between miR-147b expression and OS in patients with LUAD using Kaplan–Meier plotter. **E** Overexpression of miR-147b by transduced A549 cells compared to miR-SCR control (*n* = 3). **F** Luciferase reporter assay of 3’UTR DUSP8 transfected A549-miR-SCR vs. A549-miR-147b overexpressing cells (*n* = 3). **G** DUSP8 mRNA expression of A549-miR-147b OE vs. A549-miR-SCR transduced cells (*n* = 3). **H** Immunofluorescence staining of DUSP8 (green) counterstained with DAPI (blue), was quantified via calculation of the mean fluorescent intensity (MFI) using Fiji (*n* = 5), scale bar 50 µm. **I** Western blot analysis of DUSP8 compared to loading control ACTB (*n* = 3). **J** Volcano Plot depicting DUSP8 as top downregulated gene upon miR-147b overexpression using NanoString. Assessment of **K** colony formation, **L** proliferation, **M** migration, and (**N**) apoptosis of A549-miR-147b overexpressing cells compared to miR-SCR transduced cells (*n* = 3). Representative photomicrographs **O** of CK18 and **P** VIM antibody staining were visualized using Alexa Flour 488 coupled secondary antibody (green) and quantified via calculation of the mean fluorescent intensity (MFI) using Fiji (*n* = 5). Nuclear DNA was counterstained with DAPI (blue), scale bar 50 µm. *P*-values were determined using a two-tailed unpaired t-test with Welsh’s correction. *P*-values ≤ 0.05 were considered statistically significant for all analyses. *p ≤ 0.05, **p ≤ 0.01, ***p ≤ 0.001, and ****p ≤ 0.0001.

**Fig. 5:**
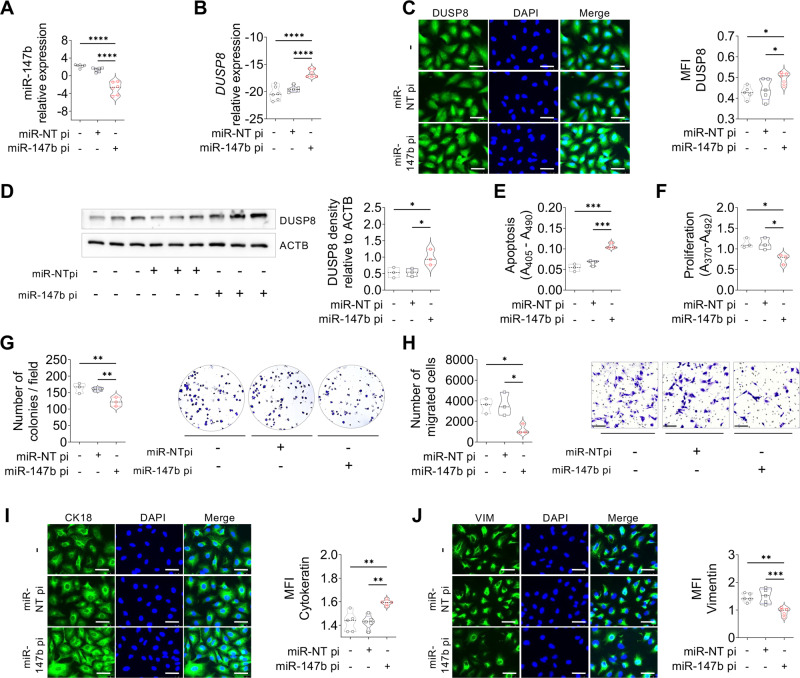
Silencing miR-147b abrogates the oncogenic potential. Validation of miR-147b and DUSP8 after treatment of A549-miR-147b OE cells with miR-non-targeting control versus miR-147b inhibitor. **A**, **B** mRNA expression of miR-147b and *DUSP8* (*n* = 6), **C** Immunocytochemistry staining of DUSP8 (green) counterstained with DAPI (blue), was quantified via calculation of the mean fluorescent intensity (MFI) using Fiji (*n* = 5), scale bar 50 µm. **D** Western blot of DUSP8 compared to loading control ACTB (*n* = 3). Inhibition of miR-147b in functional assays in vitro performed via **E** apoptosis, **F** proliferation, **G** colony formation and **H** migration (*n* = 3). Representative photomicrographs **I** of CK18 and **J** VIM antibody staining were visualized using Alexa Flour 488 coupled secondary antibody (green) and quantified via calculation of the mean fluorescent intensity (MFI) using Fiji (*n* = 5). Nuclear DNA was counterstained with DAPI (blue), scale bar 50 µm. *P*-values were determined using a two-tailed unpaired t-test with Welsh’s correction. *P*-values ≤ 0.05 were considered statistically significant for all analyses, *p ≤ 0.05, **p ≤ 0.01, ***p ≤ 0.001, and ****p ≤ 0.0001.

Growing evidence suggests that microRNAs are critical for the regulation of gene-expression networks and are frequently dysregulated in many types of cancers [17]. In this study, we performed in silico analysis of miR-binding sites for DUSP8 to identify miRNAs that may be involved in the downregulation of *DUSP8* [18]. We found that *DUSP8* has a 3’UTR binding site for miR-147b (Fig. 4A). Further in silico analysis of a TCGA LUAD cohort revealed that miR-147b expression correlates negatively with DUSP8 expression (Fig. 4B). In the same patient cohort, a significant upregulation of miR-147b in the majority of the patient samples was detected compared to non-tumor samples (Fig. 4C), associated with reduced OS (Fig. 4D). To further investigate the role of miR-147b in DUSP8 suppression, we overexpressed miR-147b in A549 and H838 cells, validated via qPCR (Fig. 4E and Supplementary Fig. S5A). Further, we performed binding assays of miR-147b to the 3’UTR sequence of DUSP8 using a dual luciferase reporter assay (Fig. 4F). A549-miR-147b overexpressing cells were found to show decreased *DUSP8* expression at the mRNA (Fig. 4G and Supplementary Fig. S5B) and protein levels (Fig. 4H, I, Supplementary Fig. S5C, D). We considered whether miR-147b targets MAPK signaling, therefore we used the NanoString approach with A549-miR-147b overexpressing cells, indeed identifying *DUSP8* as one of the most downregulated genes (Fig. 4J), the MAPK pathway as one of the most altered pathways (Supplementary Fig. S6A), alongside with downregulation of several genes (Supplementary Fig. S6B) upon miR-147b overexpression. Moreover, we could abrogate the suppression of DUSP8 by inhibiting miR-147b in A549 OE cells and H1650 cells with high miR-147b basal expression levels (Supplementary Fig. S6C). Hence, a microRNA hairpin inhibitor that binds and sequesters the mature microRNA strand was used (Fig. 5A and Supplementary Fig. S7A), resulting in restored DUSP8 expression at the mRNA (Fig. 5B and Supplementary Fig. S7B) and protein level (Fig. 5C, D, Supplementary Fig. S7C, D), which supports our previous findings.

### Upregulation of miR-147b promotes cancer progression in vitro and vice versa

To further functionally characterize the consequences of miR-147b overexpression, we assessed colony formation, proliferation and migration of A549 and H838 cells, which were all found to be significantly increased (Fig. 4K–M and Supplementary Fig. S5E–G), while apoptosis was significantly reduced (Fig. 4N and Supplementary Fig. S5H). We also observed changes in EMT marker expression (Fig. 4O, P, Supplementary Fig. S5I, J). The basal expression level of miR-147b in H838 cells was significantly lower than in A549 cells (Supplementary Fig. S6C); correspondingly, DUSP8 expression in H838 cells was high (Supplementary Fig. S2A). Further, compared to treatment with a miR non – targeting inhibitor, we could completely abrogate the pro-tumoral phenotype by adding the miR-147b inhibitor to A549-miR-147b overexpression cells and H1650 cells. Following miR-147b inhibition, apoptosis increased (Fig. 5E and Supplementary Fig. S7E) while, proliferation, colony formation and migration significantly decreased (Fig. 5F–H and Supplementary Fig. S7F–H). We also observed corresponding changes in EMT marker expression in both cell lines (Fig. 5I, J, Supplementary Fig. S7I, J).

### Overexpression of DUSP8 could rescue the tumor-supportive phenotype of A549-miR-147b OE cells in vitro

**Fig. 6:**
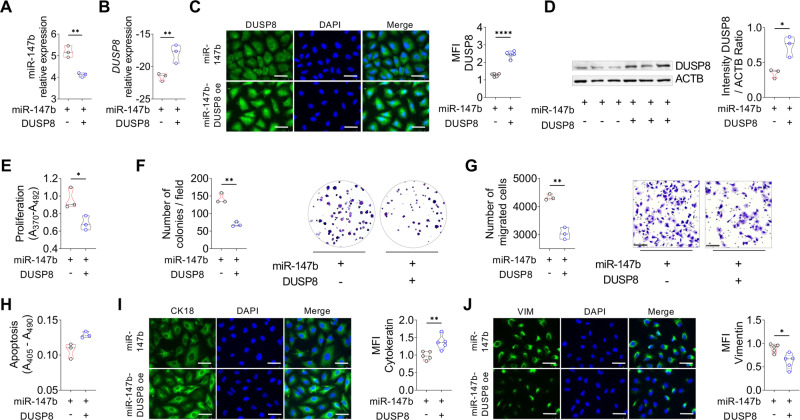
High expression of DUSP8 in A549-miR-147b overexpressing cells rescues the tumor-promoting phenotype. **A** miR-147b expression in A549-miR-147b OE cells transfected with a DUSP8 overexpressing plasmid performed by qPCR (*n* = 3). Validation of DUSP8 in A549-miR-147b-DUSP8 overexpressing cells at **B** mRNA level (*n* = 3) and **C** protein level, shown by immunocytochemistry staining of DUSP8 (green), counterstained with DAPI (blue), was quantified by calculating the mean fluorescence intensity (MFI) with Fiji (*n* = 5), scale bar 50 µm. **D** Western blot analysis of DUSP8 (*n* = 3). Quantification of **E** proliferation, **F** colony formation, **G** migration and **H** apoptosis of A549-miR-147b-DUSP8 overexpressing cells compared to A549-miR-147b transduced cells (*n* = 3). Representative photomicrographs of **I** CK18 and **J** VIM antibody staining were visualized with a secondary antibody (green) coupled to Alexa Flour 488. Nuclear DNA was counterstained with DAPI (blue), scale bar 50 µm. Quantification of the mean fluorescence intensity of CK18 and VIM (*n* = 5). *P*-values were determined using a two-tailed unpaired t-test with Welsh’s correction. *P*-values ≤ 0.05 were considered statistically significant for all analyses. *p ≤ 0.05, **p ≤ 0.01, ***p ≤ 0.001, and ****p ≤ 0.0001.

To further investigate whether we can rescue the phenotype by overexpressing DUSP8 in the A549-miR-147b OE cells, we repeated the in vitro experiments and detected diminished expression of miR-147b (Fig. 6A) and increased DUSP8 expression on RNA and protein levels (Fig. 6B–D). The functional assays depicted a suppression of cancer cell proliferation, colony formation, migration (Fig. 6E–G), no change in apoptosis (Fig. 6H) and corresponding EMT markers (CK18 and VIM) (Fig. 6I, J), predicated on DUSP8 upregulation.

### Overexpression of miR-147b suppresses DUSP8 and promotes tumor growth in orthotopic mouse models

**Fig. 7:**
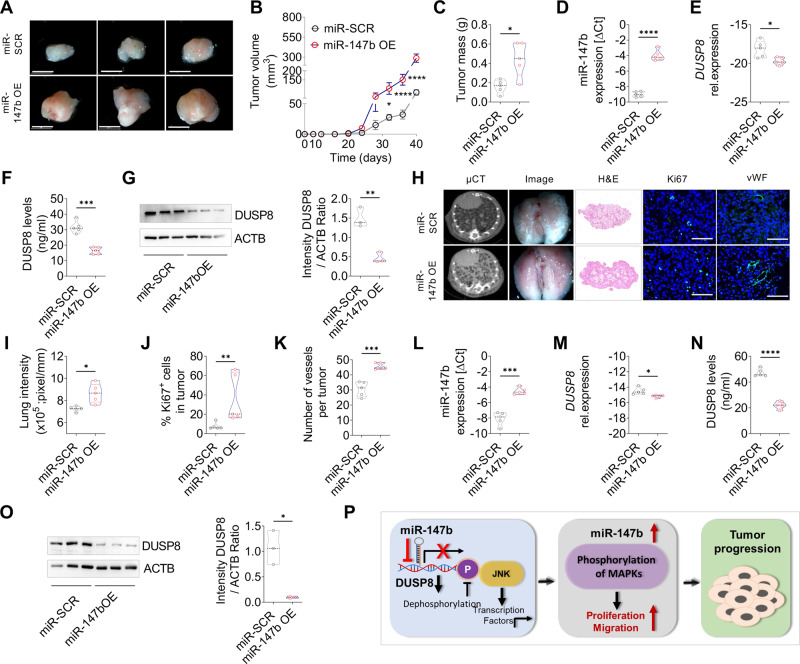
miR-147b overexpression leads to increased tumor burden in vivo. **A** Subcutaneous injection of A549-miR-SCR and A549-miR-147b cells into the right flank of immunodeficient NSG mice. Tumors were harvested after 40 days. **A** Representative macroscopic pictures of subcutaneous tumors, scale bar 5 mm. **B** Measurement of tumor size during tumor progression (*n* = 5) and **C** tumor mass after 40 days. Validation of **D** miR-147b and **E** *DUSP8* mRNA expression in mice tumor samples (*n* = 5). **F** DUSP8 level measured by ELISA (*n* = 5). **G** Western blot analysis of DUSP8 in miR-147b OE tumors compared to miR-SCR tumors (*n* = 3). **H** Intravenous injection of A549-miR-SCR and A549-miR-147b cells into the tail vein of immunodeficient NSG mice. Representative images of micro-CT scans, extracted lung images, H&E-stained sections, and immunofluorescence staining for Ki67 and vWF (green) and DAPI (blue) in tumor sections. Scale bars, 50 μm. **I** Quantification of average lung intensity (*n* = 5). Quantification of proliferating **J** Ki67^+^cells (green) (*n* = 5, 5 images per animal) and **K** vWF (*n* = 5, 5 images per animal) counterstained with DAPI (blue) within the tumor counted per high power field (HPF) using Fiji Software. Validation of **L** miR-147b and **M** *DUSP8* mRNA expression in mice lung tumor samples (*n* = 5). **N** DUSP8 level measured by ELISA (*n* = 5). **O** Western blot analysis of DUSP8 expression (*n* = 3). **P** Schematic representation of miR-147b-mediated tumor progression via suppression of DUSP8. Suppression of DUSP8 by miR-147b leads to inhibition of JNK de-phosphorylation resulting in activation of JNK signaling. Overexpression of miR-147b alters the phosphorylation of MAPKs and leads to increased proliferation and migration. The opposite effects were observed by silencing miR-147b expression and thereby restoring DUSP8 function with cancer-suppressive properties, leading to increased apoptosis. Data are shown as mea*n* ± standard error of the mean using two-way ANOVA with Bonferroni’s multiple comparisons (**B**) and two-tailed unpaired t-test with Welch’s correction (**C**–**O**). *P*-values ≤ 0.05 were considered statistically significant for all analyses, *p ≤ 0.05, **p ≤ 0.01, ***p ≤ 0.001 and ****p ≤ 0.0001.

Human A549 cells stably transfected with scrambled control miR (miR-SCR) and A549-miR-147b overexpressing cells were injected subcutaneously into the right flank (Fig. 7A) or intravenously (Fig. 7H) into NSG immunodeficient mice. In both models, the tumor burden was significantly higher in mice injected with miR-147b OE cells than in mice injected with miR-SCR (Fig. 7A–C, H, I). We further revalidated increased expression of miR-147b (Fig. 7D, L), and as expected we observed reduced *DUSP8* expression at the mRNA (Fig. 7E, M) and protein levels (Fig. 7F, G, N, O), which confirm our in vitro findings. Moreover, miR-147b overexpression significantly increased proliferation and vessel density in the lung tumor model (Fig. 7J, K). We also observed less apoptosis (Supplementary Fig. S8A, B) and changes in EMT marker expression with decreased cytokeratin and increased vimentin expressions in the tumor tissue (Supplementary Fig. S8C–F), which confirms the huge impact of miR-147b on tumor progression.

## Discussion

This study investigated the role of DUSP8 in lung cancer progression and its regulation by miR-147b, with focus on MAPK signaling. In silico analysis of human lung cancer cohorts was combined with in vitro and in vivo models of DUSP8 and miR-147b overexpression as well as silencing to decipher the interplay between the tumor suppressive potential of DUSP8 and tumor supportive potential of miR-147b in LUAD, offering as new targets for therapeutic intervention.

DUSPs have been implicated as key modulators of critical signaling pathways that are dysregulated in various diseases [8, 19]. DUSP8 belongs to a superfamily of DUSPs that includes DUSP10 and DUSP16. All three DUSPs have a more complicated domain structure than other DUSPs, and all three are localized in the cytoplasm and the nucleus [8]. In this study, we found that DUSP8 expression in samples from patients with lung cancer correlates with OS, particularly with a devastating reduction in advanced LUAD stage 3 male patients, while the gender disparity is favorable [20]. In contrast, there was no such correlation with the expression of DUSP10 or DUSP16. In addition, other cancer types (e.g., PDAC, RPCC, and CSCC) showed the same correlation between DUSP8 expression and survival as LUAD. While DUSP genes appear to be transcriptionally inducible, DUSP8 also has basal expression levels in the heart, brain, lung, and colon [21]. Until now, the role of DUSP8 in the progression of cancer has been largely neglected. By using a phosphokinase assay, we were able to identify potential new substrates GSK-3α/β, GSK-3β, Src, STAT5a/b, WNK1, PRAS40, RSK1/2, β-catenin, c-Jun and HSP60 known as drivers of cancer, whose relevant residues could be dephosphorylated by DUSP8 in addition to the reported DUSP8 relevant residues of JNK and p38 [21, 22]. Our in silico analysis of genes associated with JNK signaling revealed that OS is decreased in lung cancer when downregulated. Specifically, decreased expression of MAPK8 correlates negatively with patient survival and DUSP8 expression. Furthermore, publicly available data depicted a downregulation of JNK proteomics and phosphoproteomics phosphosite levels in LUAD patient samples, indicating further involvement in dephosphorylation by other phosphatases of the DUSP family [7, 14]. In addition, the downregulation of JNK-upstream molecules also affects clinical outcome. However, JNK may play contradictory roles in promoting cell survival and proliferation and cell death [23]. Our results showed that overexpression of DUSP8 increased protein levels in the cytoplasmic fractions of A549 and H1299 cells, but did not alter the phosphorylation of JNK upstream MAPK signaling molecules. In addition, overexpression of DUSP8 significantly reduced the ability of colony formation, proliferation and migration in two different cell lines but did not significantly affect apoptosis, indicating an interference with nuclear translocation of JNK and further activation of transcription factor targets [24]. Consistent with our results, Ding et al. reported that overexpression of DUSP8 significantly suppressed the proliferation and migration of colon cancer cells in vitro [11].

We also found that inhibition of DUSP8 by siRNA in two different cell lines changed their phenotype towards proliferative and migratory features, with concomitant upregulation of EMT marker expression. Previous studies reported that DUSPs contribute to EMT in breast cancer and glioblastoma [25, 26], suggesting that DUSP8 may also play a role in these processes.

Interestingly, silencing of DUSP8 in H838 cells did not affect apoptosis at all, probably due to the high basal expression level of DUSP8, which inhibits JNK activation. It was reported that transient JNK activation promotes cell survival, while prolonged JNK activation induces cellular apoptosis [27].

To more closely examine the contribution of DUSP8 to tumor suppression, we used a xenograft model, finding significantly reduced cancer cell proliferation and tumor size as well as vessel formation and EMT marker change upon DUSP8 overexpression. Further, activation of JNK was reduced in the subcutaneous DUSP8-overexpressing tumor tissues, supporting the notion that DUSP8 may be important in lung cancer progression via regulation of JNK MAPK signaling.

To address the question of how DUSP8 is downregulated in lung cancer, we considered that post-transcriptional regulation via microRNAs may be one of many ways [21]. Using an online miRNA binding prediction tool, we found that DUSP8 has a 3’-UTR binding site for miR-147b, which inhibits translation or degradation of DUSP8 mRNA as shown by qPCR and luciferase reporter assay. Interestingly, DUSP8 expression correlates negatively with miR147b, which is consistent with low patient survival. In addition, Ding et al. reported that DUSP8 is also a target gene of miR-21, which plays a role in colorectal cancer [11]. To further investigate the role of miR-147b in lung cancer progression, we overexpressed this miRNA in two different lung cancer cell lines. As expected, DUSP8 was found to be downregulated at the mRNA and protein levels. To further support these findings, we used the NanoString approach to assess the overall expression profile of miR-147b-transduced A549 cells. It was not surprising to find that DUSP8 was among the most downregulated genes and that the MAPK pathway and the associated genes were markedly altered by miR-147b overexpression. Functional analysis of the two lung cancer cell lines revealed a strong tumor-supportive phenotype with increased colony formation, proliferation and migration, as shown in previous lung cancer-related studies [28, 29]. Due to the suppression of DUSP8 by miR-147b and the inhibition of JNK activation, we observed a significant decrease in apoptosis in lung cancer cell lines. This may be due to the fact that mir-147b may inhibit both upstream and downstream signaling of JNK [30]. Further, the tumor cells obtained a mesenchymal phenotype upon miR-147b overexpression, suggesting a role in the process of EMT. To date, several miRNAs have been identified as critical regulators of EMT [31]. Using a miR-147b hairpin inhibitor, we demonstrated complete abrogation of the pro-proliferative phenotype in A549-miR-147b OE and H1650 cells. Recent studies showed similar results by miR-147b silencing in H1975 cells and thyroid carcinoma cell lines [29, 32]. In addition, we were able to rescue the tumor suppressive phenotype in A549-miR-147b OE cells via overexpression of DUSP8, confirming the impact of miR-147b mediated inhibition as shown previously [28]. Further in vivo experiments reported significantly increased tumor burden in subcutaneous and lung tumors with miR-147b overexpression. In both models, we observed decreased levels of DUSP8 and less phosphorylated JNK, confirming our in vitro results.

In conclusion, our results demonstrate strong lung cancer suppressive properties of DUSP8 in vitro and in vivo, and a corresponding supportive role of miR-147b as an inhibitor of DUSP8 expression in lung cancer progression. Via its interaction with DUSP8 and thereby major MAPK signaling cascades, miR-147b promotes tumor cell proliferation and migration, inhibits cancer cell apoptosis and enhances EMT signatures, thus offering a novel target for therapeutic intervention (Fig. 6P).

## Methods

### Cell culture

A54, -cells (ATCC CRM-CCL-185, American Type Culture Collection, Manassas, VA, USA) were cultured in Dulbecco’s Modified Eagle Medium Nutrient Mixture F12 (Gibco, Life Technologies, Carlsbad, CA, USA) containing 10% fetal bovine serum (FBS; Gibco) and 1% penicillin-streptomycin (10,000 U/mL, Gibco). H838 cells (ATCC CRL-5844, American Type Culture Collection) and H1650 cells (ATCC CRL-5883, American Type Culture Collection) were cultured in Roswell Park Memorial Institute medium 1640 (Gibco) containing 10% of FBS and 1% of penicillin-streptomycin (10,000 U/mL, Gibco). All cell lines were cultured at 37 °C under 5% CO_2_ and passaged every 2-3 days when reaching confluence. The cell line was authenticated by the manufacturer and checked for mycoplasma, using LookOut^®^ Mycoplasma PCR Detection Kit to guarantee all cells were mycoplasma-free.

### DUSP8 overexpression and siRNA knockdown

A549, A549-miR-147b OE and H1299 cells were seeded in a 96-well plate (5 ×104 cells/well) and incubated for 24–48 h for the overexpression of DUSP8. Regarding transfection, 200 μL of a transfection solution containing 1 µg of DUSP8-plasmid (RC208337, OriGene Technologies, Rockville, MD, USA), 6 μL of Fugene HD transfection reagent (Promega, Madison, WI, USA), and Opti-MEM reduced serum medium (Gibco) was prepared. After 24 h, the medium was changed to the culture medium of each cell line, to which 10 μL/mL of G418 (Gibco) was added for selecting successfully transfected cells. The cell number subsequently increased, and the cells were assayed. Regarding siRNA transfection, 1 ×10^5^ A549 and H838 cells were seeded in 6-well plates and incubated for 48 h. Thereafter, the cells were transfected with ON-TARGETplus human DUSP8 siRNA or non-targeting control siRNA (Horizon, Cambridge, UK) using DharmaFECT transfection reagent (GE Healthcare, Chicago, IL, USA), according to the manufacturer’s instructions. Finally, the cells were harvested and assayed after 48–72 h of incubation.

### miR-147b lentiviral transduction and stable overexpression

HEK293T cells were co-transfected with the lentiviral overexpressing the envelope plasmid pMD2.G and the packaging plasmid pCMV∆R8.2 (both Addgene, Watertown, MA, USA) using Fugene HD (Promega, Madison, WI, USA) and reduced medium. After 24 h, the medium was replaced with complete medium, and viral particles were harvested. After an additional 24 h, the viral particles were used to transfect A549 cells with a final concentration of 0.8 mg/mL of polybrene (Merck, TR-1003-G; Darmstadt, Germany) twice after 6 h. 0,02% Puromycin (A1113803, Gibco) was used to select and culture the transduced A549 cells. Lentiviral constructs for miR-SCR lentiviral non-targeting control (VSC11714), and shMIMIC-miR-147b (GSH11926-213625996) were purchased from Horizon Discovery Ltd. (Cambridge, UK). The lentiviral vectors were also used for stable transfection of H838 cells. Here cells were seeded in a 6 well-plate (1 ×10^5^ cells/well). For transfection, 200 µL of a transfection solution containing 1 µg plasmid, 6 µL Fugene HD transfection reagent, and Opti-MEM reduced serum medium per well was prepared. After 24 h, the medium was changed to each cell line’s culture medium, containing 0,02% Puromycin for selection of positive cell clones. Subsequently, the cell number was then increased under selection pressure and the cells were assayed.

### miRNA loss of function

For miRNA-147b loss of function studies, A549 and H1650 cells were treated with miRCURY LNA hsa-147b miRNA power inhibitor or the respective non-targeting control miR (Qiagen, Hilden, Germany) by adding the power inhibitor directly to the confluent cells with a final concentration of 50 nM. The cells were incubated for 48–72 h before being assayed.

### Colony formation assay

Approximately 300 cells per well of each cell clone were seeded in triplicate in a 6-well plate and incubated in 10% FBS medium for 7 days. The cells were then washed with phosphate-buffered saline (PBS). After fixation in methanol for 3 min, the cells were stained using 10% crystal violet (Sigma-Aldrich, St Louis, Missouri, USA) for 5 min. Images were taken, and the colonies were counted using ImageJ’s cell counter (National Institutes of Health [NIH], Bethesda, MD, USA).

### Proliferation and apoptosis assay

Tumor cells (A549, H838, H1299 and H1650) were seeded at 5000 cells/well in a 96‐well plate and incubated in the appropriate growth medium for 24 h. The next day, proliferation was assessed using a bromodeoxyuridine (BrdU) cell proliferation assay kit, and apoptosis was assessed using an ELISA‐based cell death detection kit (both Roche, Basel, Switzerland), according to the manufacturer’s protocol. Colorimetric signals were detected using a microplate reader (Tecan, Männedorf, Switzerland) to measure the optical density (OD) at 370 nm and 492 nm for proliferation and at 405 nm and 490 nm for apoptosis.

### Migration assay

To perform cell migration assay, 5 ×10^5^ cells/well of each cell clone were resuspended in FBS-free medium and seeded in 24-well cell culture inserts (Falcon, Corning, New York, NY, USA). The lower chamber was filled with medium containing 10% FBS. After a cell-specific incubation time (A549: 5–6 h, H838: 16 h, and H1299: 16 h, H1650: 16 h), the inner side of the membrane of the cell culture inserts was cleared from cells using a cotton swap. The membranes were then fixed with methanol for 3 min and stained with crystal violet for 10 min (Sigma-Aldrich). The membranes were cut out using a scalpel, placed on a slide, and, covered with Pertex mounting medium (Medite Service AG, Dietikon, Switzerland). The slides were scanned using NanoZoomer slide scanner (Hamamatsu Photonics, Hamamatsu, Japan). We quantified the number of migratory cells per membrane using ImageJ software with the Fiji plug-in an image-based tool for counting nuclei (National Institutes of Health, Bethesda, MD, USA). Four technical replicates were conducted for each experiment.

### Protein extraction and western blot analysis

Cells were lysed with RIPA lysis buffer (SCBT, sc-24948, Santa Cruz Biotechnology, Dallas, TX, USA) supplemented with a complete protease inhibitor cocktail (Roche, 11697498001, Roche, Basel, Switzerland), phenylmethylsulfonyl fluoride (Sigma- Aldrich, 93482-250ML-F, St. Louis, MO, USA), and sodium orthovanadate (NEB, P0758S, New England Biolabs, Frankfurt, Germany). Tumor tissue was disrupted with ceramic beads, and cell lysates were centrifuged to remove cell debris. Protein concentration was measured before dilution and denaturation by heat and treatment with 2-mercaptoethanol (Carl Roth, 4227.1, Carl Roth, Karlsruhe, Germany). Protein samples were mixed with 5x SDS sample application buffer and boiled for 5 min and were separated by sodium dodecyl sulfate-polyacrylamide-gel-electrophoresis (Bio-Rad Laboratories, Hercules, CA, USA) and blotted on a polyvinylidene difluoride-membrane (Bio-Rad Laboratories) and incubated overnight at 4 °C with primary antibodies. The following primary antibodies were used: DUSP8 (1:1000, Novus Biologicals #31169, 1:1000, A9113 Antibodies.com, Stockholm, Sweden), mouse β-actin (1:5000, ab6276) from Abcam (Cambridge, UK), SAPK/JNK (1:1000 #9252), phospho-SAPK/JNK (1:1000, #4668), ASK1 (1:1000, #3762), phospho-ASK1 (1:1000, #3764), SEK1/MKK4 (1:1000, #9152), phospho-SEK1/MKK4 (1:1000, #9156), MKK7 (1:1000, #4172) and phospho-MKK7 (1:1000, #4171) from Cell Signaling Technology (Danvers, MA, USA). Subsequently, the membranes were washed in TBST and incubated for 2 h with a corresponding horseradish peroxidase (HRP)-conjugated secondary antibody, either anti-rabbit HRP IgG conjugate (1:3000; Promega, Madison, WI, USA) or anti-mouse HRP IgG conjugate (1:3000, Promega). The resultant chemiluminescence signals were detected after treatment with Western Bright Sirius chemiluminescent substrate (Advansta, Menlo Park, CA, USA) using IBright (Thermo Fisher, Waltham, MA, USA).

### DUSP8 ELISA

Concentrations of DUSP8 in lysates were determined using a human DUSP8 ELISA Kit (abx511194, Abbexa, Cambridge, United Kingdom), according to the manufacturer’s instructions. The DUSP8 level was measured in duplicates using a microplate reader (Tecan, Männedorf, Switzerland).

### Luciferase reporter assay

We performed a luciferase reporter assay using a Dual-Glo luciferase assay system (Promega, E2920) according to the manufacturer’s instructions. In brief, cells were co-transfected with the firefly luciferase plasmid negative control vector (CmiT000001-MT06), miRNA 3’UTR β-actin (HmiT016381-MT06), or miRNA 3’UTR DUSP8 (HmiT088501-MT06; GeneCopoeia, Rockville, MD, USA) and Renilla luciferase plasmid negative control vector (CmiT000001-MT06) was used as the internal control. For the generation of mutant 3’UTR binding sites the the Q5® Site-Directed Mutagenesis Kit (E0554) (NEB, Ipswich, MA, US) and primer 3’UTR mutant (forward) ATGCCAGTGCGCCGCGCCCTGTTC and (reverse) GAAGAACTGCAGAGAGTTC were used according to manufactures instructions. The plasmids were transfected into A549-miR-SCR and A549-miR-147b OE cells in accordance with the manufacturer’s instructions and incubated for 48 h. Subsequently, cells were lysed with passive lysis buffer and transferred to white 96-well plates. Luciferase assay reagent II was used, and firefly luciferase was measured using an Infinite M200 PRO microplate reader (Tecan, Männedorf, Switzerland). STOP and Glo reagents were used, and Renilla luciferase was measured to normalize the activity of firefly luciferase.

### Immunohistochemistry (IHC) and immunofluorescence staining

In preparation for IHC staining, 3-µm tissue sections were rehydrated, and antigen-retrieval was performed with citrate buffer (Life Technologies, Darmstadt, Germany) as described in a previous study [33]. Sections were then blocked and incubated overnight with rabbit anti-DUSP8 antibody (1:1000; NBP1-88385) from Novus Biologicals (Centennial, CO, USA), CD45 monoclonal Antibody (CD45-2B11, Thermo Fisher, Waltham, MA, USA) and anti-pan cytokeratin antibody [C-11] (ab7753, Abcam, Cambridge, UK) as primary antibodies, followed by mmPRESS® Duet Double Staining Polymer Kit (HRP – anti-rabbit IgG-brown, AP – anti-mouse IgG-magenta) (MP-7714-15) (vector laboratories, Burlingame, CA, US) for detection according to the manufacturer’s instructions. Slides were counterstained with hematoxylin (AppliChem GmbH, Darmstadt, Germany) and embedded with Entellan (Merck, Darmstadt, Germany). Pictures were taken using NanoZoomer slide scanner (Hamamatsu Photonics, Japan). For immunofluorescence staining, paraffin-embedded sections were rehydrated and antigen retrieval was performed with citrate buffer as described above. In brief, slides were incubated for 2 h with the following primary antibodies: Ki67 (1:1000, ab15580), Cyt18 (1:800, ab181597), Vim (1:1000, ab8978) from Abcam (Cambridge, UK), and vWF (1:900, #A0082) from Dako (Agilent Technologies, Santa Clara, CA, USA), followed by washing steps and 1-h incubation with an Alexa Flour 488 coupled secondary antibody (Life Technologies, Darmstadt, Germany). The nuclei were counterstained using Immunoselect antifading mounting medium DAPI (Dianova, Hamburg, Germany). All the sections were stored at 4 °C and images were taken at constant exposure obtained by fluorescent microscopy (Leica, Wetzlar, Germany) at 40× magnification.

### Animal experiments

NOD.Cg-Prkdcscid Il2rgtm1Wjl/SzJ (NSG) female mice were purchased from Charles River Laboratories (Sulzfeld, Germany). A549-EV and A549-DUSP8-OE cells or A549-miR-SCR and A549-miR-147b OE cells (1 ×10^6^) were randomly injected subcutaneously into the right flank of 6–8-week-old NSG mice and A549-miR-SCR and A549-miR-147b OE were intravenously injected into the tail vein as described in a previous study [34]. The animals were sacrificed after 40 days and their tumors were harvested and immersed in formalin for paraffin sections or in Tissue-Tek for cryosections for histological examination. Subcutaneous tumor volume was calculated based on caliper measurements using the modified ellipsoidal formula (L × W2)/2. The maximal tumor size of 1500 mm^3^ was not exceeded. The tumor volume in lung tumor-bearing mice was measured using high-resolution micro-CT (SkyScan 1276, Bruker, Billerica, MA, USA). Image reconstruction was done with image reconstruction software (NRecon, v1.6, Micro Photonics), and tumor burden is presented as average lung intensity calculated with ImageJ (NIH) through manual segmentation of metastatic tumors as previously described [35].

### Study approval

All experiments using animal models were performed according to the German Law for Animal Protection and the National Institute of Health Guidelines for Care and Use of Laboratory Animals, and this study was approved by the appropriate local authorities (Regierungspräsidium Darmstadt, Hessen, Germany; study approval number: B2/1202). The study protocol for human tissue donation was approved by the ethics committee (“Ethik Kommission am Fachbereich Humanmedizin der Justus Liebig Universität Giessen”) of the University Hospital Giessen (Giessen, Germany) in accordance with national law and with “Good Clinical Practice/International Conference on Harmonisation” guidelines. Written informed consent was obtained from each patient or the patient’s next of kin (reference AZ 58/15).

### Supplementary methods

Additional experimental procedures, including acquisition of human tumor data, miRNA target prediction, q-PCR, NanoString data, Immunofluorescence staining, TUNEL assay, subcellular protein fractionation and phospho-kinase array are available in the Supplementary information.

### Statistical tests

Sample size was estimated based on previous experience with the experimental approaches. For all tumor models at least 5 mice per group are required for growth analysis and tumor weight/burden without exclusion. For proliferation, vessel counts, CK18, VIM and TUNEL at least 5 mice (5–7 images per mice) are required. For immunocytochemistry stainings at least 5 randomized taken images per group were analyzed. For in vitro assays e.g. proliferation, migration, colony formation and apoptosis, each experiment was independently repeated at least 3 times with at least 3 replicates, with similar results. All statistical analyses were performed using GraphPad Prism 9 software (GraphPad Inc., San Diego, CA, USA). One-way analysis of variance (ANOVA), followed by Tukey’s multiple comparison tests was used to compare the means of more than two independent groups; two independent groups. Two-tailed student’s t-test with Welsh’s correction was used to compare two independent groups. Two-way ANOVA with Bonferroni’s multiple comparison test was used to evaluate the effect of two grouping variables (e.g., tumor progression over time). Data are expressed as mean ± standard error of the mean. Statistical significance was set at *P* < 0.05.

### Supplementary information

Supplementary text and figure

## Data Availability

All relevant data are available from the authors upon request.
