# Supplementary material for: miR-147b mediated suppression of DUSP8 promotes lung cancer progression
Source: Oncogene. 2024 Feb 23;43(16):1178–89. doi: 10.1038/s41388-024-02969-7 (PMC11014796; doi:10.1038/s41388-024-02969-7)
Supplement: Supplementary file 1 — Supplementary text and figure [file 41388_2024_2969_MOESM1_ESM.docx]

**Supplementary material**

**miR-147b mediated suppression of DUSP8 promotes lung cancer progression**

Kati Turkowski^1,2^, Frederik Herzberg^1^, Stefan Günther^1^, Andreas Weigert^3,4^, Tamara Haselbauer^1^, Ludger Fink^5^, David Brunn^1^, Friedrich Grimminger^2,6^, Werner Seeger^1,2,6^, Holger Sültmann^7^, Thorsten Stiewe^2,8^, Soni S. Pullamsetti^1,2,6^, Rajkumar Savai^1,2,4,6^*

**Affiliations:**

^1^Max Planck Institute for Heart and Lung Research, Member of the German Center for Lung Research (DZL), Member of the Cardio-Pulmonary Institute (CPI), Bad Nauheim, 61231, Germany.

^2^Institute for Lung Health (ILH), Justus Liebig University, 35392, Giessen, Germany

^3^Goethe-University Frankfurt, Faculty of Medicine, Institute of Biochemistry I, Germany

^4^Frankfurt Cancer Institute (FCI), Goethe University, and German Cancer Consortium (DKTK), Germany

^5^Institute of Pathology and Cytology, UEGP, Wetzlar, Germany

^6^Department of Internal Medicine, Member of the DZL, Member of CPI, Justus Liebig University, 35392, Giessen, Germany

^7^Cancer Genome Research Group, German Cancer Research Center (DKFZ), German Center for Lung Research (DZL), and German Cancer Consortium (DKTK), Heidelberg, Germany

^8^Institute of Molecular Oncology, Philipps-University, 35043 Marburg, Germany.

***Corresponding author:** [rajkumar.savai@mpi-bn.mpg.de](mailto:rajkumar.savai@mpi-bn.mpg.de)

[savai.rajkumar@innere.med.uni-giessen.de](mailto:savai.rajkumar@innere.med.uni-giessen.de)

**Supplementary Materials and Methods**

**Supplementary References**

**Supplementary Figures S1 - S8**

**Supplementary Materials and Methods**

**Acquisition of human tumor data.**

We analyzed 513 LUADs that had been profiled using mRNA RNA-seq and miRNA analysis (www.kmplot.com) for Kaplan–Meier analysis ^1^. The expression levels of DUSP8, DUSP10, DUSP16, MAPK8, MAPK9 and miR-147b were categorized as low or high based on the median ^1^. The dataset used to compare the expression levels of DUSP8, MAPK8 and MAPK9 expression in lung tumor tissues versus non-tumor tissues was obtained from the UCSC Xena system as DESeq2 standardized and includes GTEx and TCGA data ^2^.

Proteomics and phosphoproteomics data were obtained from LinkedOmics database https://linkedomics.org^3^

**MicroRNA target gene prediction.**

The mature sequences of miR-147b were obtained from the miRbase (<http://www.mirbase.org/>) database. The miRNA binding site prediction was performed using the <https://cm.jefferson.edu/rna22/Interactive/> RNA22 v2 microRNA target detection tool.

**Real-time polymerase chain reaction (qPCR).**

Total RNA isolation was performed using RNeasy Mini Kit (Qiagen, Hilden, Germany) according to the manufacturer’s protocol. Subsequently, 1000 ng of total RNA was transcribed into cDNA using a high-capacity cDNA reverse transcription kit (Thermo Fisher Scientific, 4368813) according to the manufacturer’s instructions. qRT-PCR was performed for diluted cDNA and analysis was performed using a StepOnePlus Real-Time PCR System (Thermo Fisher Scientific) with PowerUp SYBR Green Mastermix (A25778, Thermo Fisher Scientific) at the following conditions: 2 min at 50°C, 2 min at 95°C, followed by 40 cycles of 15 seconds at 95°C and 1 min at 60°C. All primer pairs were subjected to a BLAST search with the NCBI primer BLAST tool (https://www.ncbi.nlm.nih.gov/tools/primer-blast/) and purchased from Sigma-Aldrich (St. Louis, MO, USA). The following primers were used: 18S: forward (5’-GGCCCTGTAATTGGAATGAGTC-3’), reverse (5’-CCAAGATCCAACTACGAGCTT-3’); *DUSP8* forward (5’GTCCCCATCAACGACAACTAC-3’), reverse (5’-CAGTGGACGATGACTTGGCAG-3’. The *18S* rRNA gene was used as the housekeeping gene. The delta cycle (dCT) was calculated as follows: mean (CT (18S)) – mean (CT (GOI)). For detection of miRNA expression, cDNA was synthesized using 5 ng of miRNA-enriched total RNA using miRCURY LNA™ universal cDNA synthesis kit (Qiagen, Hilden, Germany) according to the manufacturer’s instructions. The synthesized cDNA was diluted 60× and used for the qRT-PCR analysis of candidate miRNAs using a miRCURY LNA SYBR® green PCR kit (Qiagen, Hilden, Germany) and hsa-miR-147b miRCURY LNA Primer and has-miR-103a-3p (GeneGlobe, Qiagen, Hilden Germany) according to manufacturer’s instructions. The specificity of the miRNA amplification was evaluated by melting curve analysis. The has-miR-103a-3p LNA primer was used as the reference gene primer. Data were analyzed using the StepOne software v2.3 and normalized to the expression of housekeeping gene 18sRNA. Further, the level of mRNA expression was represented as ΔCt values (i.e., Ct value of the housekeeping gene – Ct value of the gene of interest).

**NanoString data.**

RNA from A549-SCR and A549-147b overexpression cells were evaluated for gene expression using the nCounter PanCancer Pathway panel (NanoString Technologies, Seattle, WA, USA), which interrogates 770 genes from 13 canonical pathways and selected reference genes. All normalizations and visualizations of data were performed using nSolver software (version 2.5).

**Immunocytochemistry.**

Immunocytochemical analyses were performed by seeding 1 × 10^4^ cells/well in an 8-well chamber slide (Sarstedt Inc., Nürnbrecht, Germany), followed by incubation for 24–48 hours before staining. In brief, the cells were fixed for 15 minutes by using 4% paraformaldehyde, washed with PBS (Gibco), treated with Triton-X for 15 minutes, and blocked with 1% bovine serum albumin (BSA)-PBS solution for 30 min. The primary antibodies DUSP8 (1:300, Novus Biological #31169), cytokeratin (CK18) (1:200, ab52948), and vimentin (VIM) (1:200, ab8978) from Abcam (Cambridge, UK) were diluted in 1% BSA/PBS solution and incubated with the cells for 2 hours. After washing the cells with 1% BSA/PBS solution for 15 minutes, Alexa Flour 488 goat anti-rabbit (A11008 Invitrogen, Thermo Fisher Scientific, Waltham, MA, USA) was used as the secondary antibody, and the cells were incubated for 90 minutes while being protected from light. Thereafter, the chambers were removed, and the slide was washed three times with PBS. The nuclei were counterstained using Immunoselect antifading mounting medium DAPI (Dianova, Hamburg, Germany). All the slides were stored at 4°C, images were acquired at constant exposure, and images were obtained by fluorescent microscopy (Leica, Wetzlar, Germany).

**Phospho-kinase array.**

The array was performed and cell extracts were prepared using the Human Phospho-Kinase Array Kit (R&D Systems, ARY003C, Minneapolis, MN, US) following the manufacturer’s instructions. Briefly, the diluted cleared cell lysates (400 μg) were incubated overnight following multiple washing to remove any unbound protein. Subsequently, membranes were incubated for 2 hours with the detection antibody cocktail followed by washing and 30 minutes incubation with Streptavidin-HRP, followed by detection using the chemi reagent mix, visualized by IBright (Thermo Fisher, Waltham, MA, USA).

**Subcellular protein fractionation.**

Extraction of nuclear and cytoplasmic protein from human tumor cell lines (A549, H838, H1299, H1650, A549 -EV, A549-DUSP8 OE, H1299-EV and H1299 DUSP8 OE) was conducted using NE-PER Nuclear and Cytoplasmic Extraction Reagents (Thermo Fisher Scientific) in accordance to the manufacturer’s instructions.

**In Situ Cell Death Detection (TUNEL).**

To assess apoptotic cells in mice paraffin-embedded tissue sections we used the Click-iT™ Plus TUNEL-Assay-Kit (#C10617, Thermo Fisher Scientific, Waltham, MA, USA) according to the manufacturer’s protocol.

**Supplementary References**

1 Gyorffy B, Surowiak P, Budczies J, Lánczky A. Online survival analysis software to assess the prognostic value of biomarkers using transcriptomic data in non-small-cell lung cancer. *PLoS ONE* 2013; **8**. doi:10.1371/journal.pone.0082241.

2 Goldman M, Craft B, Brooks A, Zhu J, Haussler D. The UCSC Xena Platform for cancer genomics data visualization and interpretation Paper Introduction. *bioRxiv* 2018; : 1–16.

3 Vasaikar S V, Straub P, Wang J, Zhang B. LinkedOmics: analyzing multi-omics data within and across 32 cancer types. *Nucleic Acids Research* 2018; **46**. doi:10.1093/nar/gkx1090.

**Supplementary Figures**


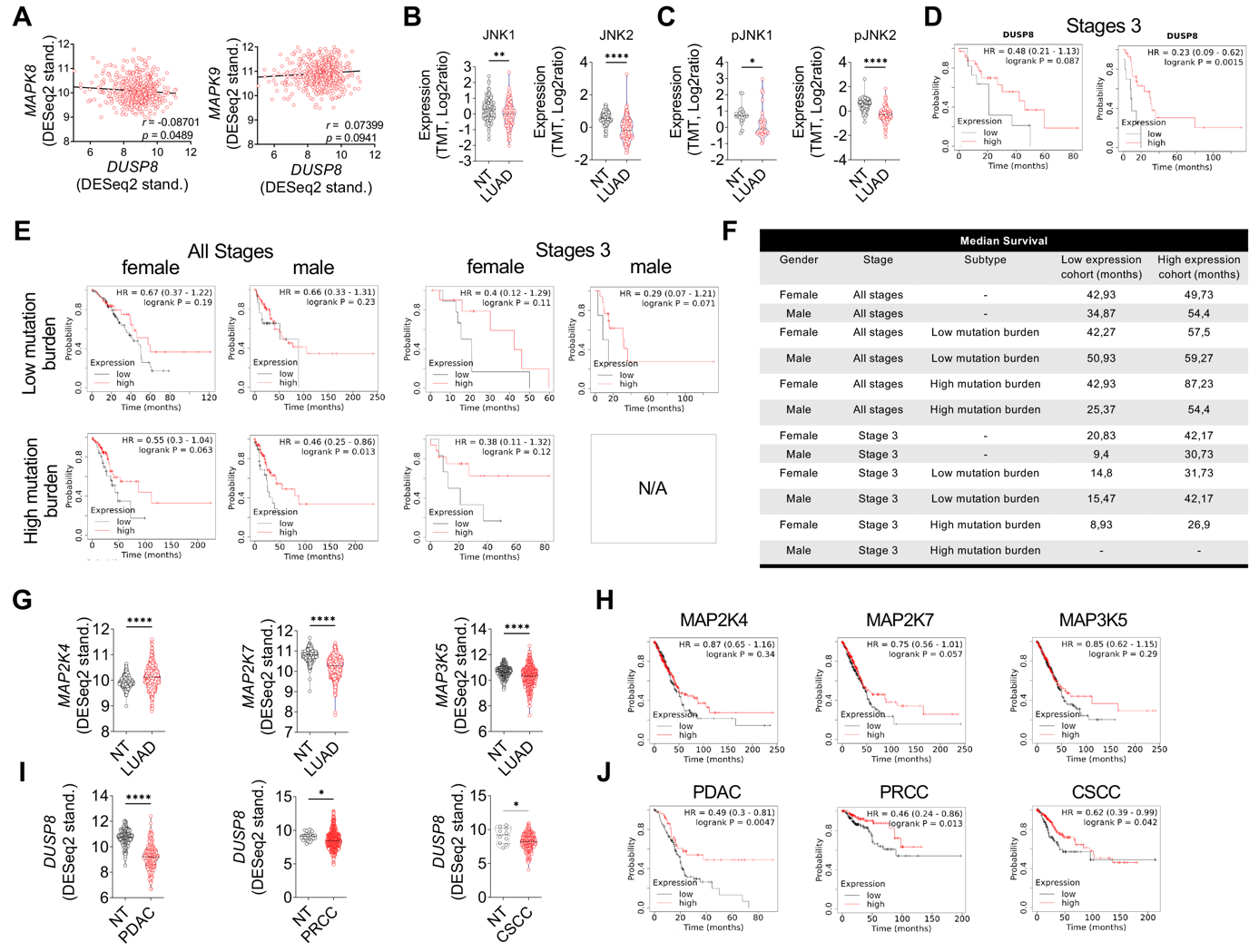


**Figure S1.** **Downregulation of DUSP8 in male LUAD patients correlated with poor OS depending on stage and mutational burden.** (**A**) Correlation between DUSP8 (n=513) expression with MAPK8 (n=513), and MAPK9 (n=513) expression. (**B**) Proteomics (gene level) and (**C**) phosphoproteomics phosphosite level data of JNK1 and JNK2 expression (**T**andem **M**ass **T**ag, log2ratio) in LUAD patients (n=110) compared to adjacent non-malignant tissue (n=101). Correlation between DUSP8 expression and OS of female (f) and male (m) in (D) stage 3 LUAD patients (f; n=79, m; n=59) and (**E**) low mutation burden (f; n=259, m; n=144), high mutation burden (f; n=155, m; n=141) of all stages and low (f; n=57, m; n=33) and high mutational burden(f; n=43, m; n/a) of stage 3 LUAD patients using KM Plotter^1^. (**F**) Table showing median survival (months) of LUAD patients dependent on DUSP8 expression, stage and mutational burden with n numbers equal to (E). (**G**) mRNA expression level of *MAP2K4*, *MAP2K7,* and *MAP3K5* in LUAD (n=513) versus NT (n=287) patient samples from TCGA TARGET GTEx cohort. (**H**) Clinical outcome of LUAD patients associated with *MAP2K4*, *MAP2K7,* and *MAP3K5* expression. (**I**) Expression of *DUSP8,* in PDAC (n=178) vs. NT (n= 165), in PRCC (n=884) vs. NT (n= 27), and CSCC (n=304) vs. NT (n=10) TCGA TARGET GTEx cohort respectively. (**J**) Clinical outcome associated with *DUSP8* expression. *P*-values were determined using a two-tailed unpaired t-test with Welsh’s correction. *P*-values ≤0.05 were considered statistically significant for all analyses, *p ≤0.05, and ****p≤0.0001.


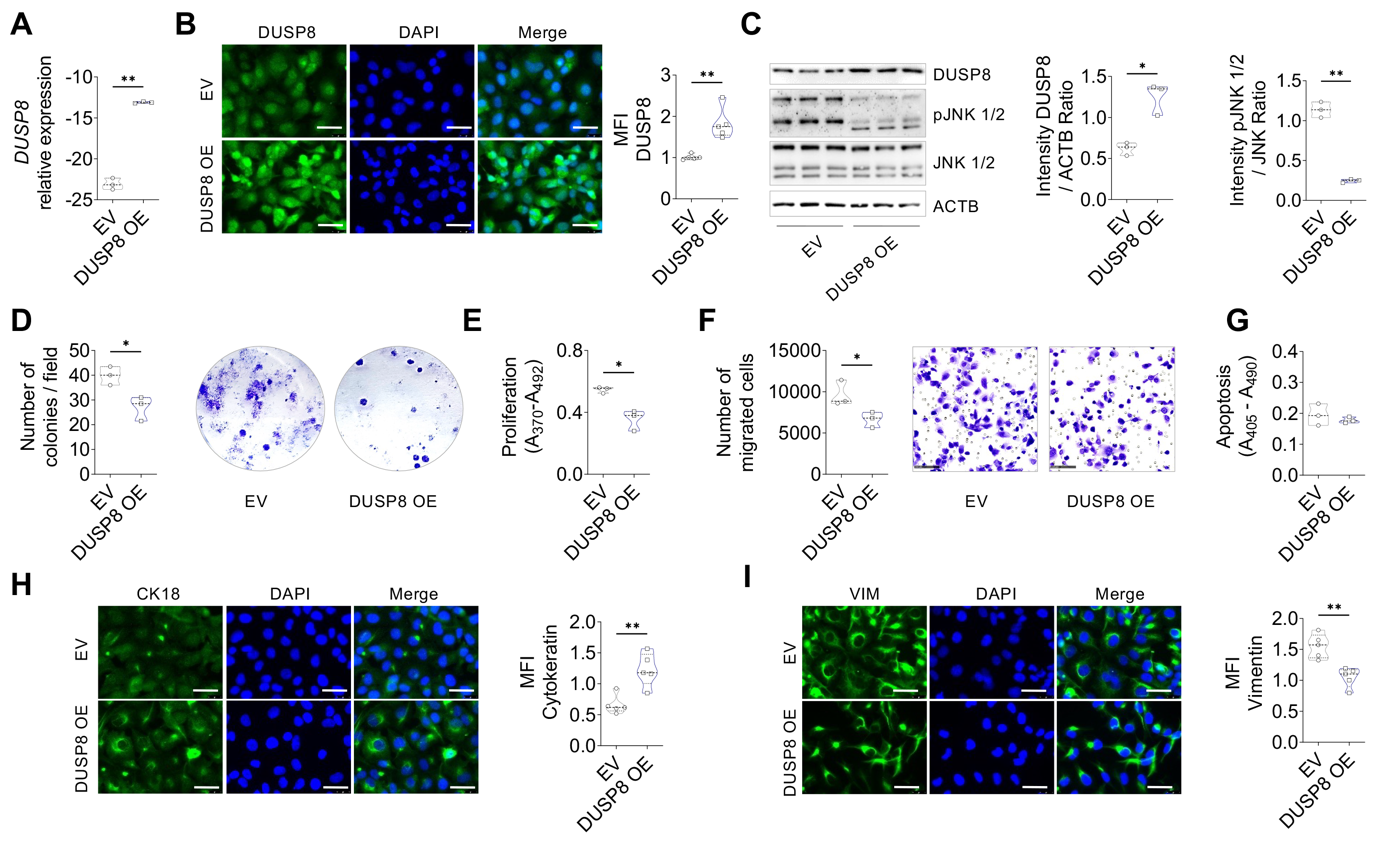


**Figure S2. DUSP8 overexpression inhibits the pro-tumoral phenotype *in vitro*.** Validation of DUSP8 overexpression after transfection of H1299 cells with empty vector (EV) and DUSP8 expression vector (OE) was quantified by (**A**) qRT-PCR (n=3), (**B**) Immunocytochemistry staining of DUSP8 (green) counterstained with DAPI (blue), was quantified via calculation of the mean fluorescent intensity (MFI) using Fiji (n=5), scale bar 50µm. (**C**) Expression of DUSP8 and phosphorylation of JNK conducted by western Blot of H1299-EV and H1299-DUSP8 OE cells (n=3). (**D**) Colony formation of DUSP8 OE cells compared with EV control cells (n=3). (**E)** Cell proliferation of DUSP8 OE cells compared to EV control cells (n=3). (**F**) The migratory ability of DUSP8 OE cells (n=3) and (**G**) apoptosis (n=3). Representative photomicrographs (**H**) of CK18 and (**I**) VIM antibody staining was visualized using Alexa Flour 488 coupled secondary antibody (green). Nuclear DNA was counterstained with DAPI (blue), scale bar 50µm. Quantification of CK18 and VIM mean fluorescent intensity (n=5). *P*-values were determined using a two-tailed unpaired t-test with Welsh’s correction. *P*-values ≤0.05 were considered statistically significant for all analyses. *p ≤0.05 **p ≤0.01 and ***p ≤0.001.


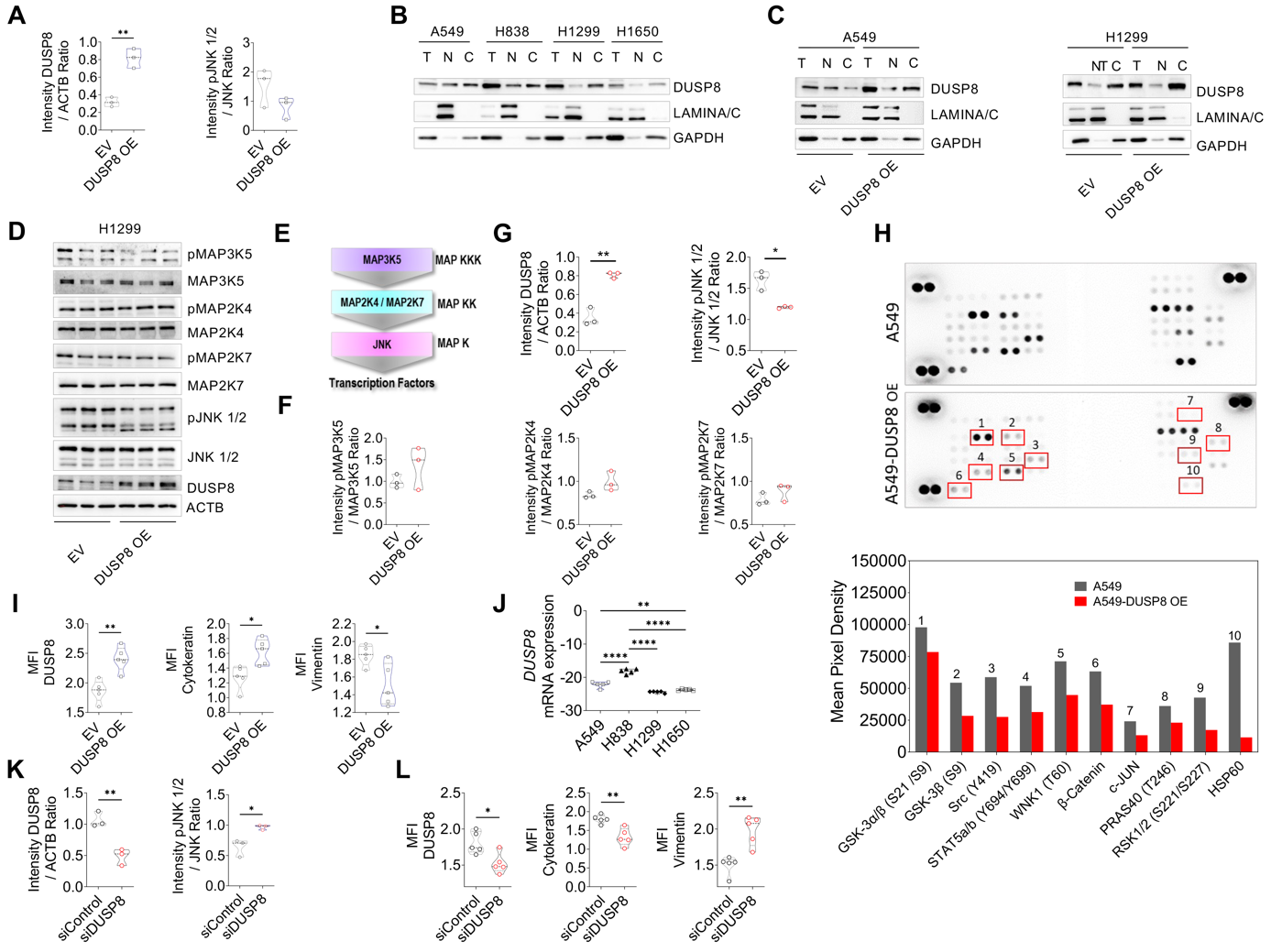


**Fig. S3 DUSP8 increased abundance in both nuclear and cytoplasmic fractions might dephosphorylate residues of oncogenic drivers in A549 DUSP8 overexpressing cells.** (**A**) Quantification of DUSP8, P-JNK and JNK protein levels in A549 DUSP8 OE cells compared to A549 EV cells (n=3). (**B**) Western blot analysis of total protein (T) nuclear (N) and cytoplasmic (C) fraction of A549, H838, H1299, H1650 cells and (**C**) A549 EV compared to A549 DUSP8 OE and H1299 EV compared to H1299 DUSP8 OE of DUSP8 expression and LAMINA/C as nuclear/cytoplasmic fractionation control and GAPDH as loading control. (**D**) Western blot analysis of MAP3K5, MAP2K4, MAP2K7 and JNK phosphorylation of H1299-EV and H1299-DUSP8 OE cells (n=3) (**E**) schematic diagram of JNK upstream signaling molecules. Quantification of bands intensity for (**F**) phosphorylated versus total protein of MAP3K5, MAP2K4, MAP2K7 and (**G**) DUSP8 to ACTB ratio and phosphorylated JNK to total JNK protein ratio (n=3). (**H**) Comparison of membranes and quantification of the pixel density of A549 control cells compared to A549 DUSP8OE cells. (n=2) (**I**) Quantification of DUSP8, cytokeratin (CK18) and vimentin (VIM) expression in A549 DUSP8 OE cells compared to EV control cells by mean fluorescent intensity (n=5). (**J**) DUSP8 mRNA expression in lung cancer cell lines (n=5) (**K**) Quantification of DUSP8, P-JNK and JNK protein levels of A549 cells after siDUSP8 silencing compared to siControl cells (n=3). (**L**) Quantification of DUSP8, CK18 and VIM expression by mean fluorescent intensity after DUSP8 silencing (n=5). Data are shown as mean ± standard error of the mean using t-test comparisons *P*-values ≤0.05 were considered statistically significant for all analyses, **p* ≤0.05 and ***p* ≤0.01.


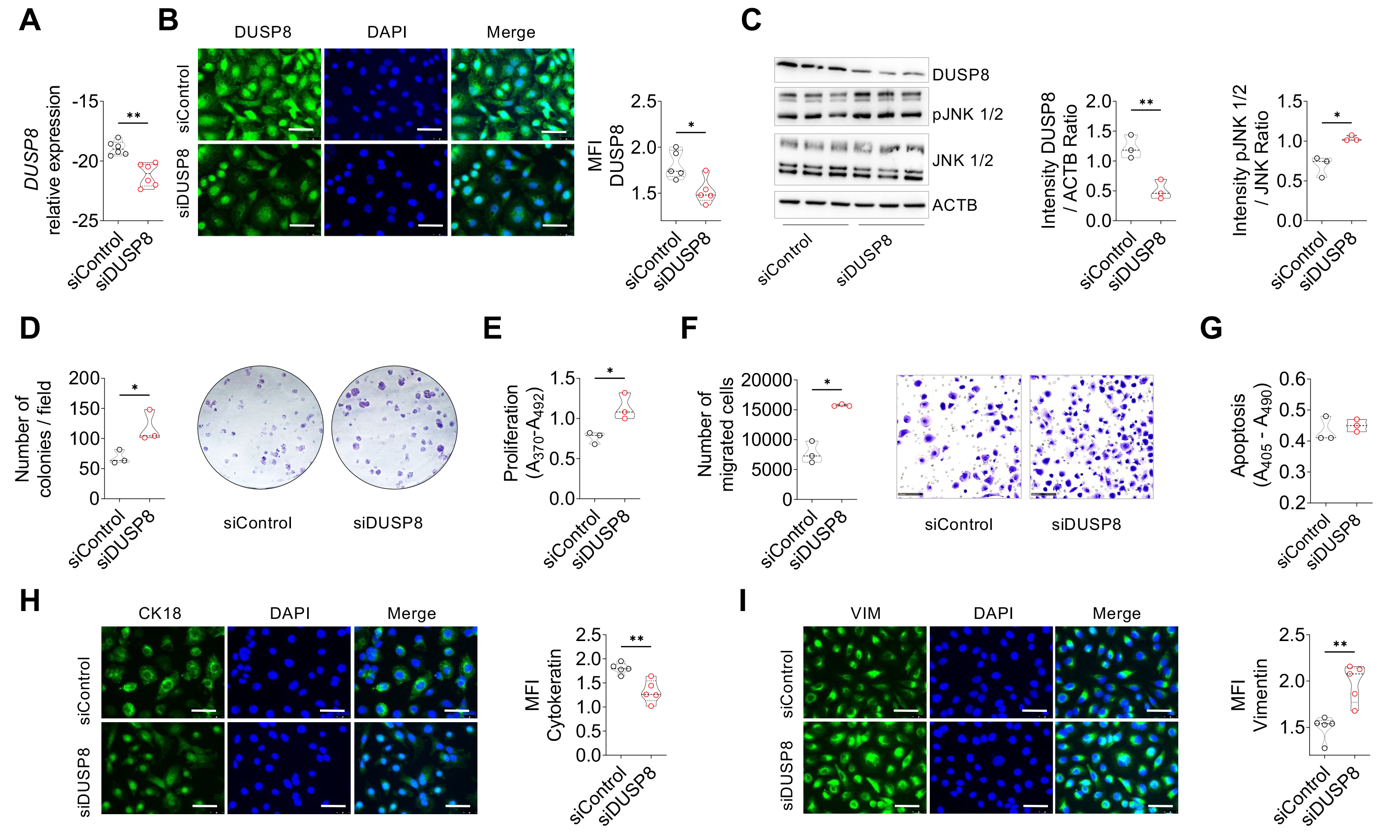


**Figure S4. DUSP8 silencing in H838 cells abrogates the tumor suppressive phenotype *in vitro*.** Validation of DUSP8 silencing in H838 cells with siRNA (siDUSP8) compared to non-targeting control (siControl) quantified by (**A**) qRT-PCR, (**B**) Immunocytochemistry staining of DUSP8 (green) counterstained with DAPI (blue), was quantified via calculation of the mean fluorescent intensity (MFI) using Fiji (n=5), scale bar 50µm. (**C**) Expression of DUSP8 and phosphorylation of JNK conducted by western Blot of H1299-EV and H1299-DUSP8 OE cells (n=3). (**D**) Colony formation of DUSP8 OE cells compared with EV control cells (n=3). (**E)** Cell proliferation of DUSP8 OE cells compared to EV control cells (n=3). (**F**) The migratory ability of DUSP8 OE cells (n=3) and (**G**) apoptosis (n=3). Representative photomicrographs (**H**) of CK18 and (**I**) VIM antibody staining was visualized using Alexa Flour 488 coupled secondary antibody (green). Nuclear DNA was counterstained with DAPI (blue), scale bar 50µm. Quantification of CK18 and VIM mean fluorescent intensity (n=5). *P*-values were determined using a two-tailed unpaired t-test with Welsh’s correction. *P*-values ≤0.05 were considered statistically significant for all analyses. *p ≤0.05 ** and p ≤0.01.


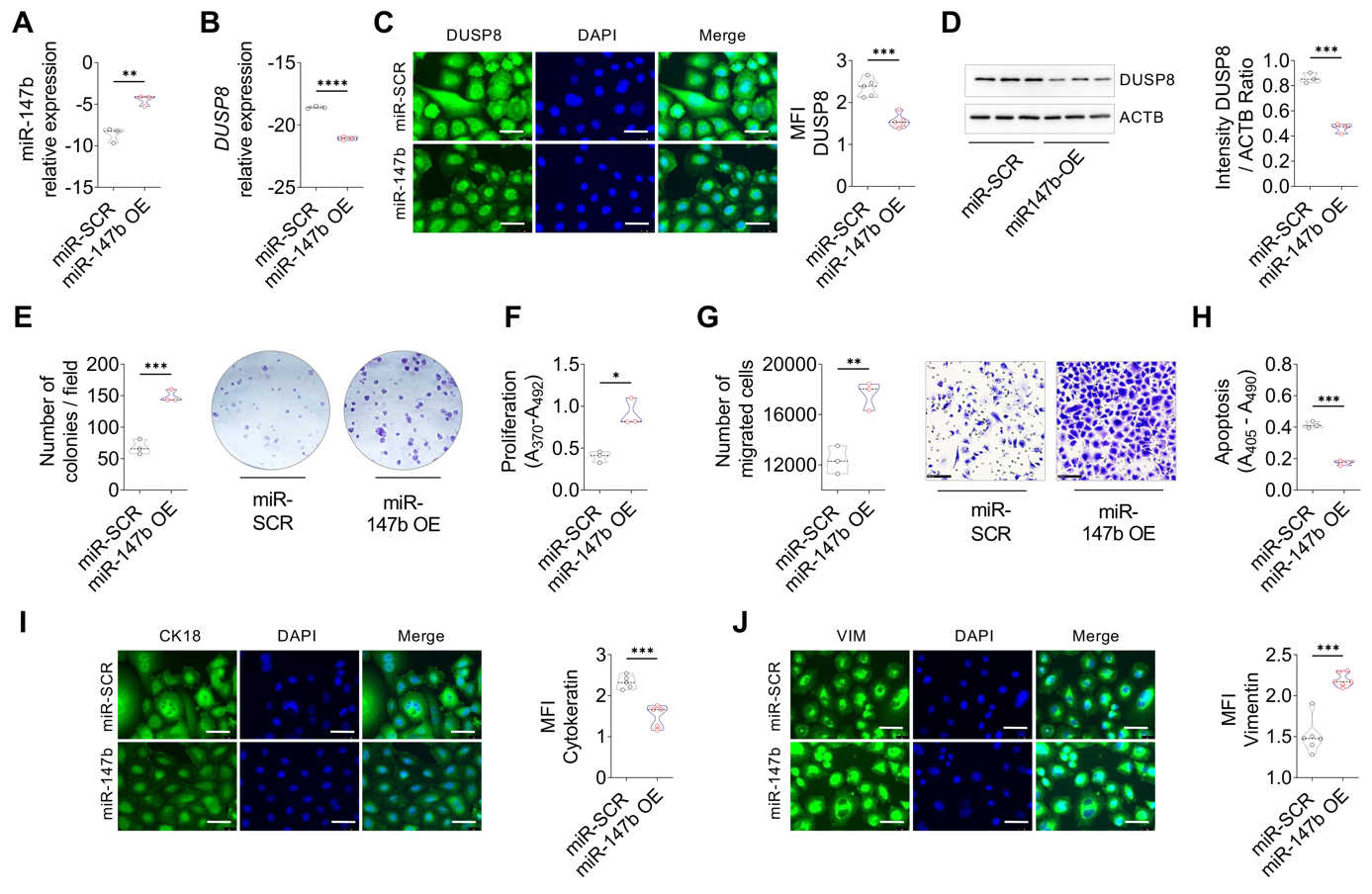


**Figure S5. High expression of miR-147b in H838 cells showed tumor-promoting phenotype *in vitro.*** (**A**) miR-147b expression in H838 transfected cells conducted via qPCR (n=3). (**B**) Validation of DUSP8 in H838-miR-147b overexpressing cells on (**B**) mRNA level (n=3) and (**C**) protein level represented by Immunocytochemistry staining of DUSP8 (green) counterstained with DAPI (blue), was quantified via calculation of the mean fluorescent intensity (MFI) using Fiji (n=5), scale bar 50µm. (**D**) Western blot analysis of DUSP8 (n=3). Quantification of (**E**) colony formation, (**F**) proliferation, (**G**) migration, and (**H**) apoptosis of H838-miR-147b overexpressing cells compared to miR-SCR transduced cells (n=3). Representative photomicrographs (**I**) of CK18 and (**J**) VIM antibody staining was visualized using Alexa Flour 488 coupled secondary antibody (green). Nuclear DNA was counterstained with DAPI (blue), scale bar 50µm. Quantification of CK18 and VIM mean fluorescent intensity (n=5). *P*-values were determined using a two-tailed unpaired t-test with Welsh’s correction. *P*-values ≤0.05 were considered statistically significant for all analyses. *p ≤0.05, **p ≤0.01, ***p ≤0.001, and ****p≤0.0001.


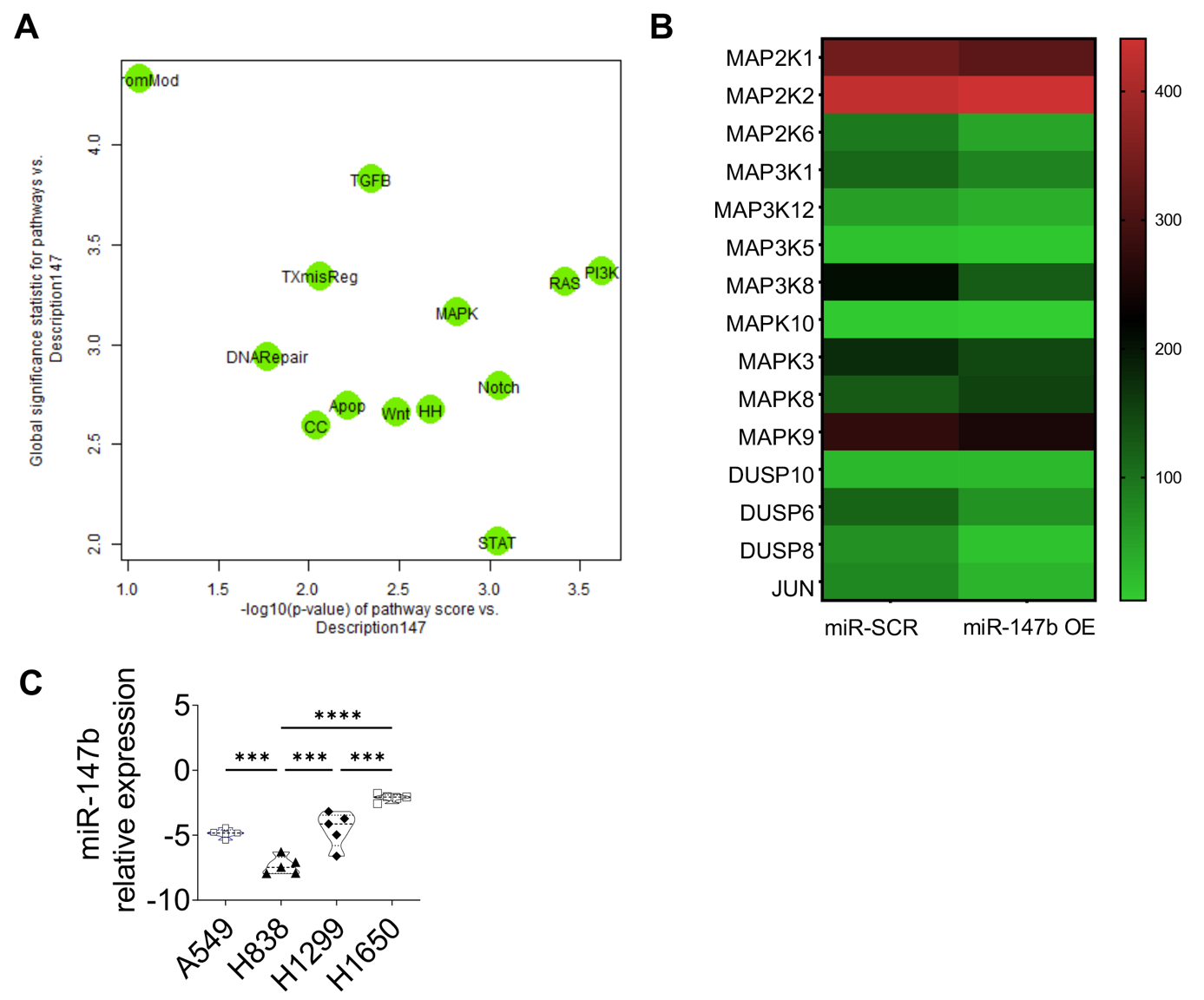


**Figure S6. NanoString analysis reveals MAPK pathway alterations associated with miR-147b overexpression.** NanoString Analysis of A549-miR-SCR compared to A549 miR-147b OE cells. **(A)** Two measures of a pathway's relationship with a covariate are displayed. The first measure summarizes the behavior of the pathway in differential expression analysis using their global significance statistics. A high global significance statistic indicates a pathway's genes are extensively differentially expressed in response to the covariate. The second measure summarizes the behavior of the pathway scores with the covariate. For each pathway score, a linear regression has been fit predicting the pathway score from the selected covariates. The horizontal axis plots each regression's -log10(p-value) for the association between its pathway score and the covariate. A high -log10(p-value) indicates a pathway score that is highly statistically significantly associated with the covariate. (**B**) Heatmap of MAPK pathway-related genes. (**C**) Expression level of miR-147b in A549, H838 H1299 and H1650 cells (n=5). *P*-values were determined using ordinary one-way ANOVA with Tukey’s multiple comparisons tests. *P*-values ≤0.05 were considered statistically significant for all analyses. *p ≤0.05, **p ≤0.01, ***p ≤0.001, and ****p≤0.0001


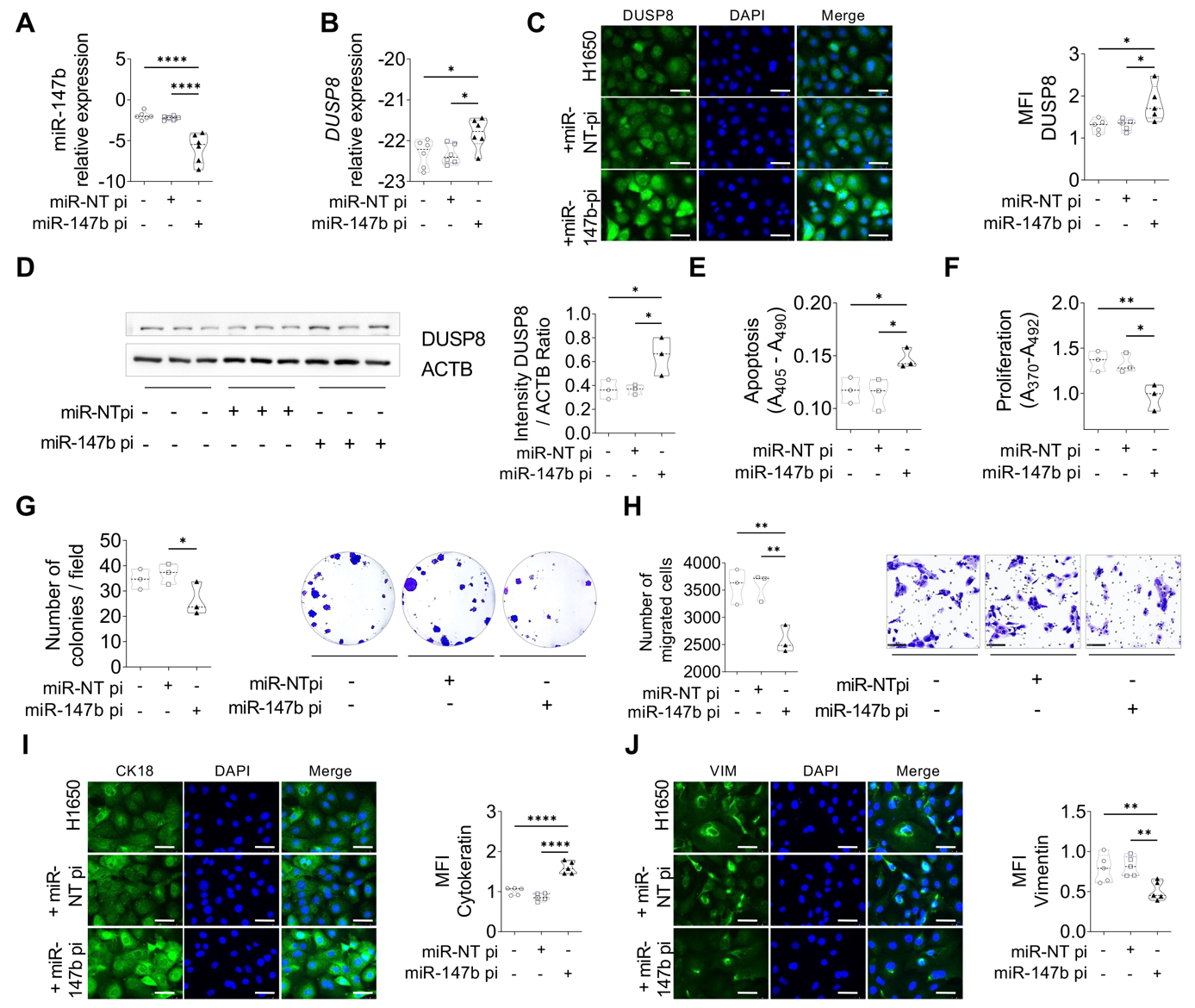


**Figure S7.** **Silencing miR-147b abrogates the oncogenic potential in H1650 cells.** Validation of miR-147b and DUSP8 after treatment of H1650cells with miR-non-targeting control versus miR-147b inhibitor. (**A, B**) mRNA expression *of* miR-147b and *DUSP8* (n=6), (**C**) Immunocytochemistry staining of DUSP8 (green) counterstained with DAPI (blue), was quantified via calculation of the mean fluorescent intensity (MFI) using Fiji (n=5), scale bar 50µm. (**D**) Western blot of DUSP8 compared to loading control ACTB (n=3). Inhibition of miR-147b in functional assays *in vitro* was performed via (**E**) apoptosis, (**F**) proliferation, (**G**) colony formation, (**H**) and migration (n=3). Representative photomicrographs (**I**) of CK18 and (**J**) VIM antibody staining was visualized using Alexa Flour 488 coupled secondary antibody (green) and quantified via calculation of the mean fluorescent intensity (MFI) using Fiji (n=5). Nuclear DNA was counterstained with DAPI (blue), scale bar 50µm. *P*-values were determined using a two-tailed unpaired t-test with Welsh’s correction. *P*-values ≤0.05 were considered statistically significant for all analyses, *p ≤0.05, **p ≤0.01, ***p ≤0.001, and ****p≤0.0001.


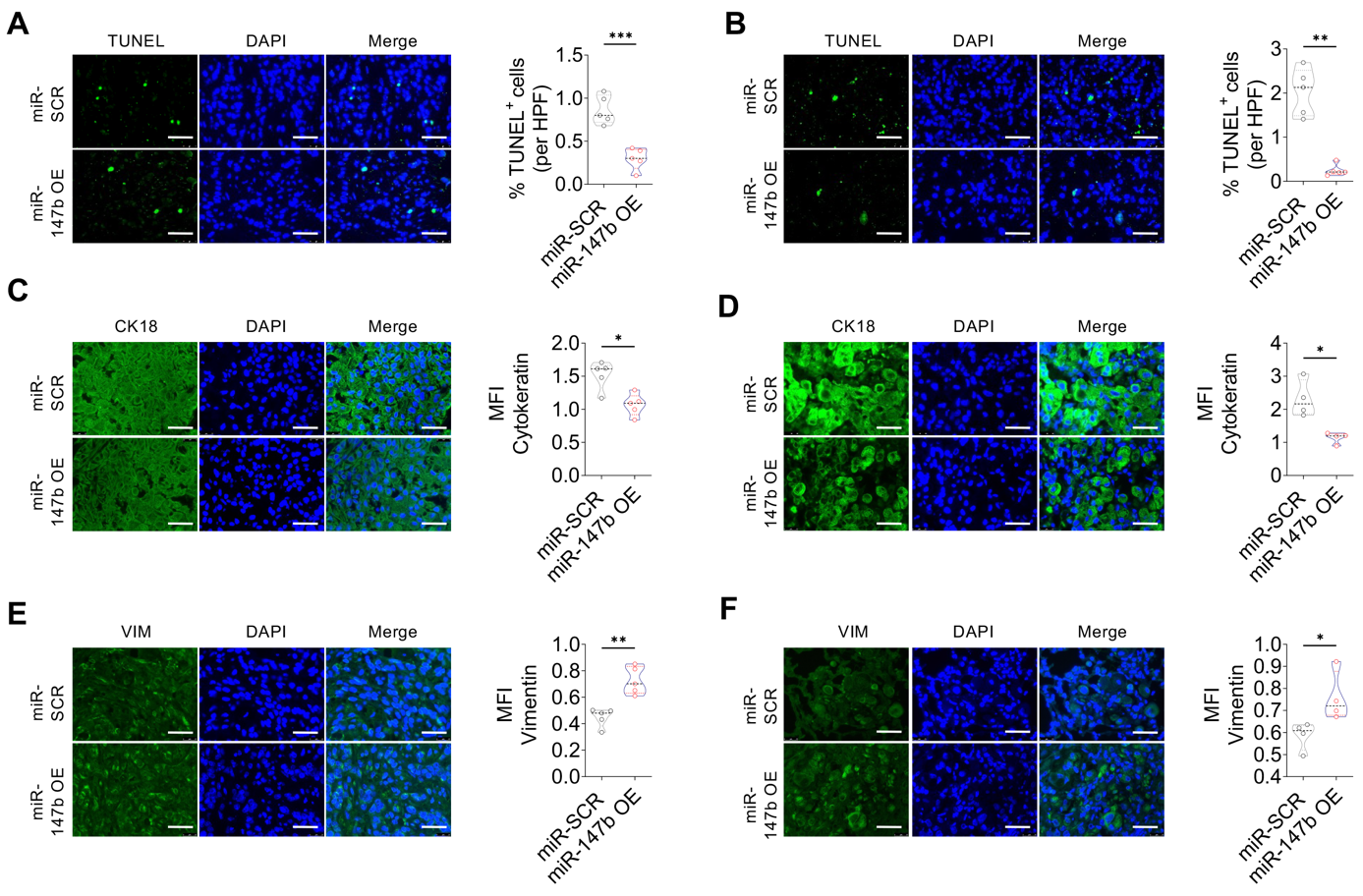


**Figure S8. Expression of cytokeratin and vimentin in miR-147b OE tumor sections.** Representative photomicrographs of TUNEL staining (green) for apoptotic cells within the **(A)** subcutaneous and **(B)** lung tumor counted per high power field (HPF) using Fiji Software (n=5, 4 images per animal). **(C-F)** Expression of CK18 and VIM by A549 miR-SCR compared with A549 miR-147b OE. Representative photomicrographs of EMT marker CK18 and VIM visualized using Alexa Flour 488 coupled secondary antibody (green). Nuclear DNA was counterstained with DAPI (blue), scale bar 50µm. Quantification of CK18 and VIM in (**C, E**) subcutaneous (n=5, 3 images per animal) and (**D, F**) lung tumors (n=4, 3 images per animal) quantified via calculation of the mean fluorescent intensity (MFI) from immunofluorescence of 5 images per animal (n=3) using Fiji. Data is shown as mean+/- standard error of the mean using a two-tailed unpaired t-test with Welch’s correction. *P*-values ≤0.05 were considered statistically significant for all analyses, *p ≤0.05, **p ≤0.01 and ***p≤0.001.
